# A Novel Protein from *Ectocarpus* sp. Improves Salinity and High Temperature Stress Tolerance in *Arabidopsis thaliana*

**DOI:** 10.3390/ijms22041971

**Published:** 2021-02-17

**Authors:** Pramod Rathor, Tudor Borza, Sophia Stone, Thierry Tonon, Svetlana Yurgel, Philippe Potin, Balakrishnan Prithiviraj

**Affiliations:** 1 Department of Plant, Food and Environmental Sciences, Dalhousie University, Truro, NS B2N 5E3, Canada; pramod.rathor@dal.ca (P.R.); Tudor.Borza@dal.ca (T.B.); syurgel@dal.ca (S.Y.); 2 Department of Biology, Dalhousie University, Halifax, NS B3H 4R2, Canada; s.stone@dal.ca; 3 Centre for Novel Agricultural Products, Department of Biology, University of York, Heslington, York YO10 5DD, UK; thierry.tonon@york.ac.uk; 4 Integrative Biology of Marine Models (LBI2M), Station Biologique de Roscoff (SBR), Sorbonne Université, CNRS, UMR 8227, 29680 Roscoff, France; philippe.potin@sb-roscoff.fr

**Keywords:** *Arabidopsis thaliana*, *Ectocarpus* sp., unknown function protein, transgenic plant, salinity, temperature, abiotic stress tolerance

## Abstract

Brown alga *Ectocarpus* sp. belongs to Phaeophyceae, a class of macroalgae that evolved complex multicellularity. *Ectocarpus* sp. is a dominant seaweed in temperate regions, abundant mostly in the intertidal zones, an environment with high levels of abiotic stresses. Previous transcriptomic analysis of *Ectocarpus* sp. revealed several genes consistently induced by various abiotic stresses; one of these genes is *Esi0017_0056*, which encodes a protein with unknown function. Bioinformatics analyses indicated that the protein encoded by *Esi0017_0056* is soluble and monomeric. The protein was successfully expressed in *Escherichia coli,* *Arabidopsis thaliana* and *Nicotiana benthamiana.* In *A. thaliana* the gene was expressed under constitutive and stress inducible promoters which led to improved tolerance to high salinity and temperature stresses. The expression of several key abiotic stress-related genes was studied in transgenic and wild type *A. thaliana* by qPCR. Expression analysis revealed that genes involved in ABA-induced abiotic stress tolerance, K^+^ homeostasis, and chaperon activities were significantly up-regulated in the transgenic line. This study is the first report in which an unknown function *Ectocarpus* sp. gene, highly responsive to abiotic stresses, was successfully expressed in *A. thaliana*, leading to improved tolerance to salt and temperature stress.

## 1. Introduction

Unfavorable abiotic stress conditions, including high salinity and temperature stress, negatively influence plant performance resulting in significant reduction of agricultural productivity. Due to climate change these stresses are becoming more severe. It has been predicted that in order to feed around 9 billion people agricultural productivity must double in the near future. Completing this demand will be challenging due to the continuous decline in the availability of water for irrigation, changing weather patterns, and the reduction of arable land area. 

In the last decades, research carried out using model plants as well as crop plants led to the identification of a large number of genes involved in abiotic stress tolerance; however, much work still remains to be performed to develop climate resilient crops. Brown algae (Phaeophyta) are multicellular organisms in the phylum heterokonts which is highly distantly related from the Archaeplastida and Opisthokonts [1]. Brown algae are among the few eukaryotic lineages which have evolved complex multicellularity [2]. These organisms have been evolving over a billion years, and during evolution, they acquired a number of distinct characteristics that are absent in the other eukaryotic lineages. For instance, phaeophytes, which originated through secondary endosymbiosis, have complex polysaccharides in their cell wall. Moreover, the secondary endosymbiosis enriched considerably the nuclear genome; as a result, brown algae display a number of distinctive metabolic pathways [3]. Brown algae are constantly subjected to high levels of abiotic stresses arising from the tidal cycles which are associated with temperature extremes, mechanical forces, and irradiation [4]. Some of the novel characteristics acquired by these organisms enabled them to survive and flourish in these harsh environmental conditions. The unique features of phaeophytes make this group interesting to explore, to decipher novel pathways and functions that very likely played essential roles in their evolutionary success; clearly, their biology is not well studied compared to that of animals and land plants. *Ectocarpus* sp. is a multicellular brown alga which has relatively small genome size (200 Mbp) in contrast to *Fucus serratus* (1095 Mbp) and *Laminaria digitata* (640 Mbp) [5,6]. *Ectocarpus* belongs to Ectocarpales, which is closely related to Laminariales, a group of seaweeds of significant economic importance [7]. Ecologically and economically, these seaweeds are of great interest because they are the source of important biomolecules such as fucoidans, laminarin and alginates. A large number of studies reported the benefits of brown algal extracts on plant health and their usage to improve agricultural productivity [8,9,10,11]. The beneficial effects include enhanced seed germination and plant establishment, improved resistance to environmental stresses, improved crop performance and enhanced post-harvest life [8,9,11]. However, many aspects of brown algal biology remain largely unknown, including the underlying molecular mechanisms of enhanced tolerance to abiotic stresses. Characterization of genes with novel functions in brown algae will advance knowledge and may lead to discovery of novel unique biomolecules which can contribute to improved, sustainable agricultural production. Complete genome sequencing of the brown algal model *Ectocarpus* sp. constituted an important step in the understanding of phaeophyte biology at the molecular level. Genome analysis followed by searches in protein databases revealed that more than 36% of the proteins were novel, being *Ectocarpus* or phaeophyte specific, with no counterpart in other taxonomic groups. These significant differences suggested that a large number of evolutionary innovations took place in this group, leading to the occurrence of many novel genes in addition to considerable divergence from homologous sequences [12]. Therefore, *Ectocarpus* genomic data represent a valuable resource with a great potential to discover novel genes and pathways involved in stress adaptation or specific bioactivities that are absent in other taxonomic groups. However, phaeophyte research is challenging, especially when the aim is to characterize and understand functions of novel genes, coding proteins of unknown function. These limitations are due to the lack of genetic resources such as methods of transformation to generate gain or loss of function mutants, or to determine subcellular localization, thus hampering most molecular studies. 

Previous transcriptomic analysis of *Ectocarpus* sp., subjected to various abiotic stresses, showed that 76% of the up-regulated genes were encoding proteins of unknown functions, with no significant similarity to any of sequences outside *Ectocarpus* sp. [13]. We report here the expression in *Arabidopsis thaliana* of the unknown function gene *Esi0017_0056* from *Ectocarpus* sp. Its expression in *A. thaliana*, using a constitutive promoter (35S) and an *A. thaliana* stress inducible promoter from the RESPONSIVE TO DESICCATION 29A gene (*RD29A*), led to improved tolerance to salinity and high temperature stress in the model plant. The protein was also successfully expressed in *Escherichia coli* and *Nicotiana benthamiana*. 

## 2. Results

### 2.1. Protein Structure, Phylogenetic Relationships and Intracellular Localization of *Esi0017_0056*

Esi0017_0056 was one of the several *Ectocarpus* sp. proteins (i.e., Esi0379_0027, Esi0154_0047, Esi0025_0042, Esi0488_0007, Esi0059_0099, Esi0322_0010, Esi0252_0035, Esi0045_0021, Esi0105_0049, Esi0182_0002, Esi0143_0016, Esi0007_0087, Esi0113_0047, Esi0538_0008, Esi0021_0137, Esi0195_0005, Esi0176_0002, Esi0266_0005 and Esi0044_0144) that were screened as potential candidates for cloning and expression in *E. coli*, *N. benthamiana* and *Arabidopsis*. All these proteins were analyzed first for the presence of transmembrane domains with HMMTOP v. 2.0 (http://www.enzim.hu/hmmtop/; accessed on 26 January 2021) and TMHMM v. 2.0 (http://www.cbs.dtu.dk/services/TMHMM-2.0/; accessed on 26 January 2021). The presence of such domains makes protein expression in any system problematic. No transmembrane domains have been predicted for Esi0017_0056. 

Homology modelling using PSIPRED provided an overall prediction of the secondary structure of this protein with a MW of 43.1 kDa and an isoelectric point (pI) of 8.74 (Supplementary Figure S1). To gain more information about Esi0017_0056 protein structure, homology-modelling was carried out using the SWISS-MODEL server. The best model (26.71% sequence identity) was built using the hypothetical protein ybiA from *E. coli* as a template. Modelling of the region between residues 132 to 291 suggested that Esi0017_0056 is well-structured with several alpha-helices and short beta-strands (Supplementary Figure S2a,b). Modelling information and data coming from similarity searches of other proteins harboring domains from the DUF1768 superfamily as well as from the automatic prediction output from PSIPRED, HMMTOP v. 2.0 and TMHMM v. 2.0 indicated that Esi0017_0056 is a soluble, monomeric protein. A full-length tertiary structure, built by DMPfold, revealed a protein with a globular structure, and additional alpha helices (Supplementary Figure S2c).

Blastp similarity searches of Esi0017_0056 in GenBank RefSeq non-redundant proteins database revealed that the closest relatives are two other proteins from *Ectocarpus*, which are also labeled as unknown conserved proteins (GenBank accession # CBJ32535 and CBJ27468). The next > 100 organisms harboring protein sequences with significant similarity (E-value: 8 × 10^−45^– 1 × 10^−37^) to these *Ectocarpus* proteins were of prokaryotic origin, including delta proteobacteria, gamma proteobacteria, firmicutes and cyanobacteria. The middle part of the 391 amino acids long Esi0017_0056 protein, that is, residues 132 to 291, was found to have strong similarity with DUF1768, a protein domain of unknown function (pfam08719). DUF1768 contains members such as *E. coli* N-glycosidase YbiA (COG3236), involved in riboflavin biosynthesis, which was initially characterized as a swarming motility protein. More recently, this family was included in the NADAR (NAD and ADP-ribose) superfamily which comprises proteins predicted to be involved in NAD-utilizing pathways, likely to act on ADP-ribose derivatives. Blastp similarity searches of Esi0017_0056 protein in GenBank RefSeq non-redundant proteins database, with restricted searches for eukaryotes, revealed that the closest eukaryotic organisms harboring a similar protein is the apusomonad *Thecamonas trahens* ATCC 50062 (E-value: 1 × 10^−38^). The organisms within this range of similarity were found to be extremely diverse, including the metazoans *Strongylocentrotus purpuratus* and *Dendronephthya gigantea*, the haptophyte *Emiliania huxleyi*, the amoebozoa *Entamoeba invadens* and the fungus *Cercospora berteroae*. The annotation of proteins in these taxa was similar to that found in prokaryotes, that is, DUF1768-domain containing protein, swarming motility protein YbiA-like and riboflavin biosynthesis protein PYRR. All these predictions refer to the same pfam08719.

To test the phylogenetic relationships of Esi0017_0056 to other similar proteins from various prokaryotes and eukaryotes lineages and to assess the likelihood that this gene occurred in *Ectocarpus* sp. through lateral gene transfer, a phylogenetic tree was generated (Supplementary Figure S3). The support for most branches was found to be weak because of the low number of conserved residues present in the DUF1768 superfamily domain. Nevertheless, analysis indicated close relationships between *Ectocarpus* sp. sequences and prokaryotic sequences (proteobacteria), and with eukaryotic apusozoan and haptophytes. Two *Ectocarpus* sp. sequences clustered together with Esi0017_0056. It is therefore likely that these *Ectocarpus* sp. sequences occurred through lateral transfer from a proteobacteria followed by duplication and divergent evolution. As *Esi0017_0056* gene contains two predicted introns, the possibility of representing a contaminant sequence of prokaryotic origin is excluded (Supplementary Figure S4).

Potential intracellular protein localization was analyzed using several prediction servers. SignalP, iPSORT and TargetP 2.0 ruled out the presence of a signal peptide or of a mitochondrial, chloroplast or thylakoid luminal transfer peptide. WoLF PSORT indicated with low, very similar scores, that the possible location of this protein can be mitochondria, cytosol or chloroplast. PSORT and DeepLoc - 1.0 predicted that the most probable location of this soluble protein is the mitochondria rather than the cytosol, but the support was not strong (Supplementary Data S1). It is worth mentioning that DeepLoc - 1.0 predicts eukaryotic protein subcellular localization using deep learning and it can differentiate between 10 different localizations: nucleus, cytoplasm, extracellular, mitochondrion, cell membrane, endoplasmic reticulum, chloroplast, Golgi apparatus, lysosome/vacuole and peroxisome. As Esi0017_0056 was predicted as a soluble protein with no obvious N- or C-terminus extensions, it is likely that in *Ectocarpus,* its translation occurs in the cytosol and in heterologous eukaryotic systems such as *A. thaliana* the process should be similar. However, we cannot formally rule out that its final destination might also depend on its interaction with other macromolecular intracellular structures. 

### 2.2. *Esi0017_0056* Protein Is Highly Expressed in E. coli, A. thaliana and N. benthamiana 

**Figure 1:**
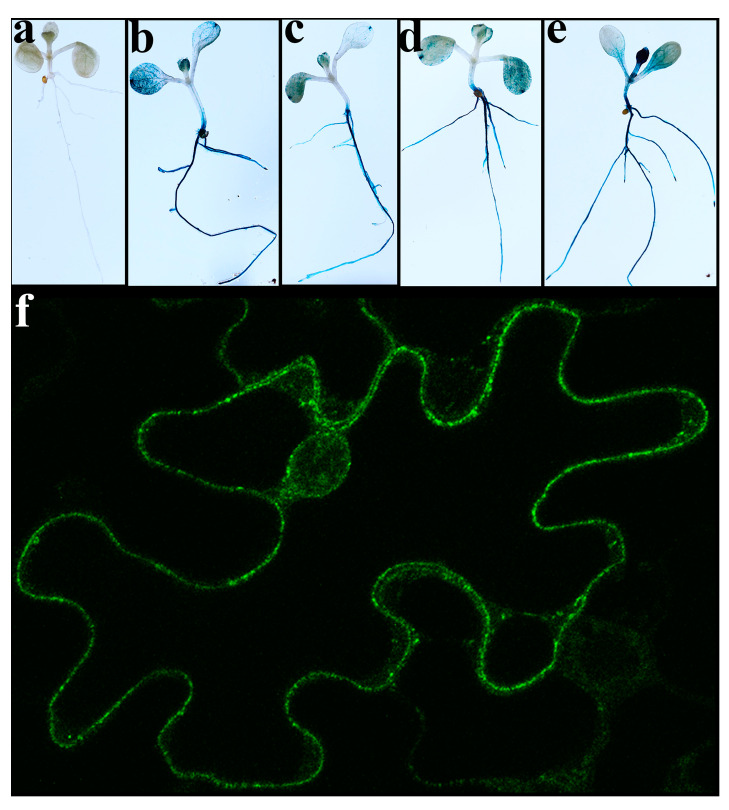
Expression of C-terminal fusion proteins *Esi0017_0056-GUS* and *Esi0017_0056-GFP*. (**a**–**e**) GUS expression in *A. thaliana* 5 days old plantlets (**a**) WT and (**b**–**e**) independent transgenic lines. (**f**) GFP expression in *N. benthamiana* leaf.

Esi0017_0056 was successfully expressed in *E. coli*; however, most of the protein was found in inclusion bodies (Supplementary Figure S5). Sufficient recombinant protein was obtained to carry out LC-MS/MS sequencing, which confirmed the expression of a full length Esi0017_0056 (Supplementary Table S1). To investigate the expression in plants the C-terminal fusion constructs of *Esi0017_0056* with GUS and GFP tags were introduced in *A. thaliana* and *N. benthamiana*, respectively. In both transgenic plants *Esi0017_0056* was found to be highly expressed (Figure 1). GUS activity staining of *A. thaliana* plantlets revealed strong occurrence in roots and many parts of the leaflets (Figure 1a–e). Confocal microscopy analysis of *N. benthamiana* leaves section expressing *Esi0017_0056*-GFP revealed strong fluorescence localized throughout the cytoplasm and around nucleus but not in the vacuole (Figure 1f). 

### 2.3. Gene Expression of Esi0017_0056 Showed That It Is Highly Expressed in A. thaliana under Standard and Salinity Stress Conditions

**Figure 2:**
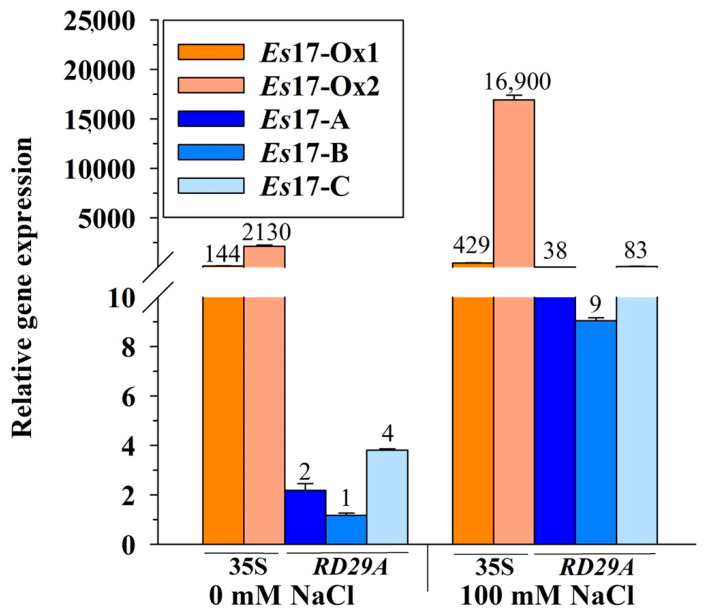
Expression of *Esi0017_0056* in two independent *A. thaliana* transgenic lines having the 35S promoter and in three independent transgenic lines having the stress inducible (RD29A) promoter under standard and salinity stress conditions. *Actin* was used as the endogenous control and transcript levels were normalized to the individual with the lowest expression from line *Es*17-B. Data represent mean ± SE from 3 biological replicates. Values listed on the bars represent relative expression, fold change ratio *vs* the line with the lowest *Esi0017_0056* expression, that is, line *Es*17-B in absence of NaCl.

To study the potential role of *Esi0017_0056* in improved tolerance to abiotic stress, the gene was cloned in two different constructs, one in which the expression was driven by the constitutive promoter 35S and the other one by the *A. thaliana* stress inducible promoter RD29A. Expression of *Esi0017_0056* was confirmed in the transgenic lines (Figure 2). In normal conditions, the expression in the *Es*17-Ox1 and *Es*17-Ox2 lines, having the 35S promoter, was much higher than that observed in *Es*17-A, *Es*17-B and *Es*17-C lines, having the RD29A promoter (Figure 2). It is worth mentioning that the expression of *Es*17-Ox lines, but not of *Es*17-A-C lines, was higher than that of *actin*, the reference gene, which is a highly expressed gene in eukaryotic systems. While Ct values of *actin* varied in the 21-22 cycles range, those of *Es*17-Ox1 and *Es*17-Ox2 were found to be around 19 cycles and 16 cycles, respectively. These results suggest that the transcription of *Esi0017_0056* in *A*. *thaliana* is efficient and the mRNA is rather stable. Upon exposure to salinity stress, the expression *Esi0017_0056* was found to be strongly up-regulated in the *Es*17-A, *Es*17-B and *Es*17-C lines (19, 9 and 20 times, respectively), while in the *Es*17-Ox1 and *Es*17-Ox2 lines these changes were found to be less pronounced (3 and 7.9 times, respectively) (Figure 2). The increase amounts of transcripts in the 35S lines upon exposure to salinity stress might be due to the stability of *Esi0017_0056* mRNA, process observed in other systems as well [14].

### 2.4. Expression of Esi0017_0056 Exhibited Better Tolerance to Salinity Stress in A. thaliana Seedlings and Plants

**Figure 3:**
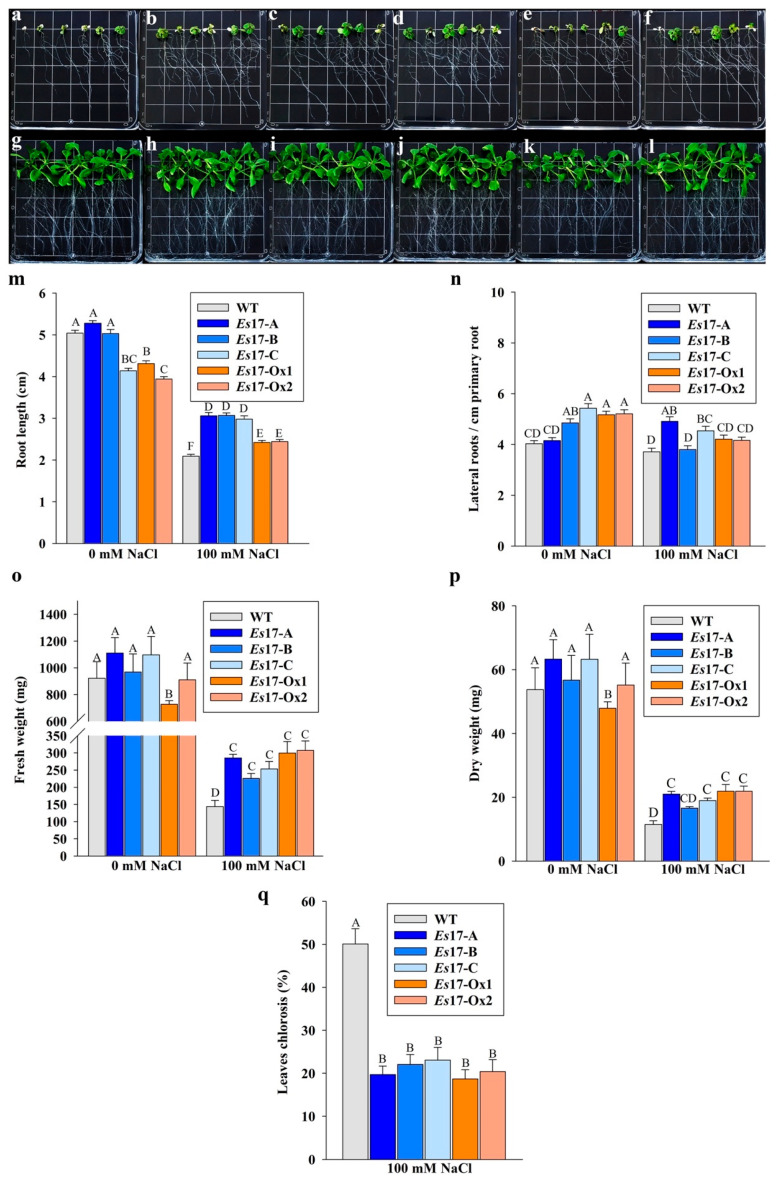
Growth of wild type and 2 independent 35S promoter (*Es*17Ox-1-2,) and of 3 independent stress inducible promoter (*Es*17A-C) transgenic *A. thaliana* seedlings, expressing *Esi0017_0056* in presence and absence of 100 mM NaCl. In plates (**a**–**f**), seedlings were grown under salt stress conditions while in plates (**g**–**l**), seedlings were grown in standard conditions. (**a**,**g**) WT, (**b**,**h**) *Es*17-A, (**c**,**i**) *Es*17-B, (**d**,**j**) *Es*17-C, (**e**,**k**) *Es*17-Ox1, (**f**,**l**) *Es*17-Ox2, (**m**) root length, (**n**) number of lateral roots per cm of primary root, (**o**) fresh weight, (**p**) dry weight and (**q**) leaf chlorosis. Values represents mean and standard error (*n* = 90) for root length, lateral roots, leaf chlorosis and *n* = 9 for fresh and dry weight. Means and SE followed by the same letter are not significantly different. The plants were photographed at 9 days after transfer in standard and under salinity stress conditions.

To examine the effects of *Esi0017_0056* expression on salinity stress tolerance of transgenic lines, the seedlings were exposed to 100 mM NaCl. After one week of exposure to 100 mM NaCl, transgenic seedlings had significantly longer roots, higher number of lateral roots per cm of primary root, reduced leaf chlorosis and higher biomass as compared to the wild type seedlings (Figure 3). 

**Figure 4:**
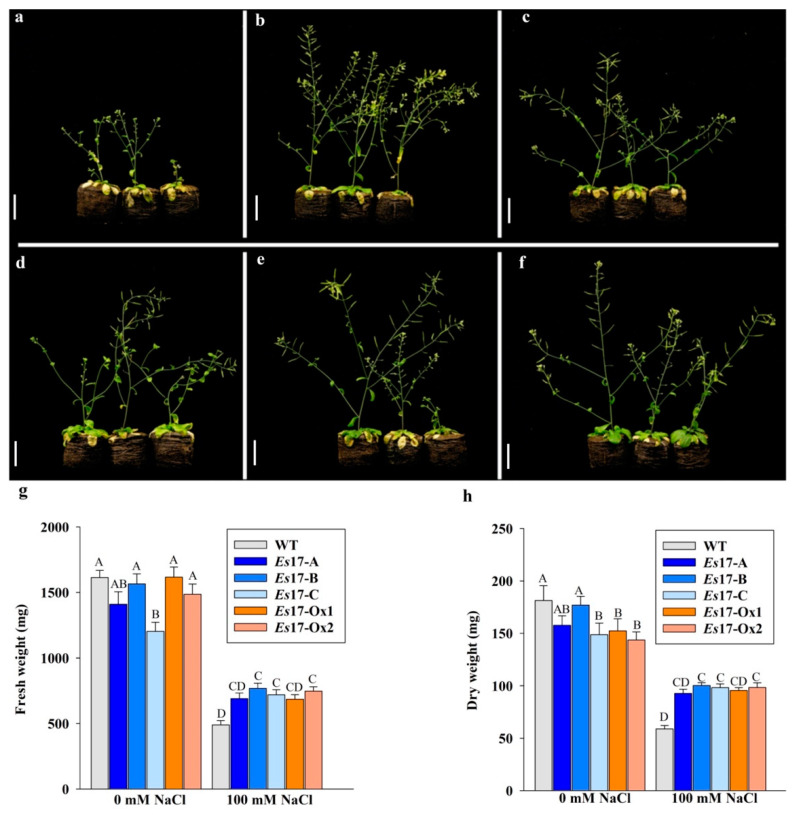
Growth of wild type and 2 independent 35S promoter (*Es*17Ox-1-2,) and of 3 independent stress inducible promoter (*Es*17A-C) transgenic *A. thaliana* plants, expressing *Esi0017_0056*, in presence of 100 mM NaCl concentration maintained throughout the experiment. (**a**) WT, (**b**) *Es*17-A, (**c**) *Es*17-B, (**d**) *Es*17-C, (**e**) *Es*17-Ox1, (**f**) *Es*17-Ox2, (**g**) fresh weight and (**h**) dry weight. The plants were photographed at 20 days after irrigation. Values represent mean and standard error (*n* = 15). Means and SE followed by the same letter are not significantly different.

In order to verify if the enhanced stress tolerance observed in seedlings can be observed in plants grown in soil, 15-days-old transgenic plants were irrigated with 200 mM NaCl, and concentrations of around 100 mM were maintained in the peat pellet. After one week of exposure to salinity stress, wild type plants exhibited symptoms of growth retardation and leaf chlorosis whereas transgenic plants grew better (Figure 4). Analysis of biomass data suggested that transgenic plants had significantly higher fresh weight and dry weight as compared to the wild type plants (Figure 4). When the effect of promoters (constitutive and stress-inducible) was contrasted, transgenic lines generated using stress inducible promoter showed slightly reduced fresh weight under standard conditions as compared to the wild type plants, while the lines generated using constitutive promoter showed slightly reduced dry weight. Overall, none of the transgenic plants grew better than the wild type plants under standard conditions, if both fresh weight and dry weight are considered (Supplementary Figure S6).

### 2.5. Expression of Esi0017_0056 Exhibited Enhanced Tolerance to High Temperature Stress in A. thaliana Seedlings

To examine the effects of *Esi0017_0056* expression on high temperature stress tolerance of transgenic lines, the seedlings were exposed to 40 °C for 24 h. One week after the high temperature stress, the seedlings of transgenic lines recovered much faster and showed better growth, including significantly higher fresh and dry weight, when compared to the wild type seedlings (Supplementary Figure S7). 

### 2.6. A. thaliana Plants Expressing Esi0017_0056 Showed Reduced Electrolyte Leakage 

**Figure 5:**
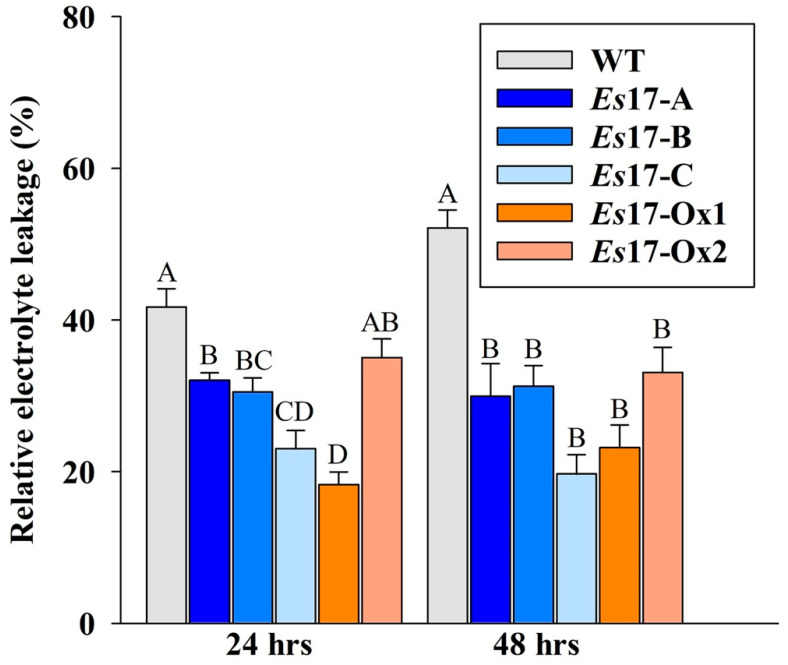
Electrolyte leakage of wild type and 2 independent 35S promoter (*Es*17Ox-1-2,) and of 3 independent stress inducible promoter (*Es*17A-C) transgenic *A. thaliana* plants, under 100 mM salinity stress conditions. Values represent mean and standard error (*n* = 15). Means and SE followed by the same letter are not significantly different.

To estimate the effect of salinity stress on membrane stability, electrolyte leakage was measured at 24 and 48 h after the exposure to salinity stress of transgenic plants grown on peat pellets. Both 35S and stress inducible promoter transgenic lines showed significant reduction in leakage of electrolytes compared to the wild type plants, indicating higher membrane stability in transgenic plants (Figure 5).

### 2.7. A. thaliana Plants Expressing Esi0017_0056 Exhibited Altered Expression of Stress Responsive Genes

**Figure 6:**
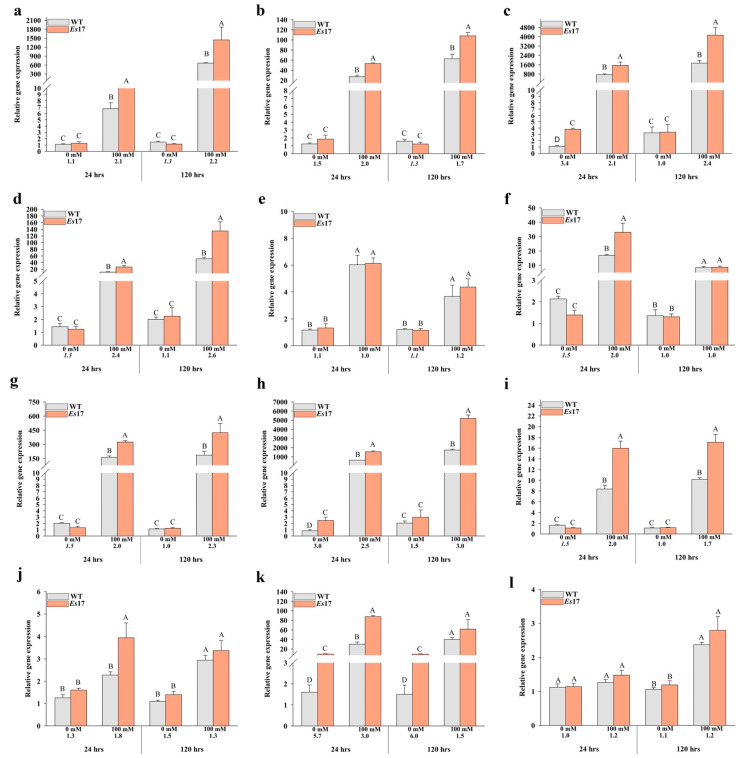
Gene expression analysis of stress inducible marker genes in wild type and transgenic *A. thaliana* plants (*Es*17Ox-2) grown in absence and presence of 100 mM NaCl. Two time points (24 and 120 h) were studied. *Actin* was used as the endogenous control, and transcript levels were normalized to the individual with the lowest expression. Values listed under the bars represent fold difference; default font values represent up-regulation while italicized font values represent down-regulation. Data represent mean ± SE from 3 biological replicates. (**a**) *DREB2A,* (**b**) *RD29A,* (**c**) *RD29B,* (**d**) *RD26,* (**e**) *RD22,* (**f**) *RD20,* (**g**) *RAB 18,* (**h**) *LEA,* (**i**) *LEA14,* (**j**) *NHX1,* (**k**) *HSP70* and (**l**) *HSFA1D.* Means and SE followed by the same letter are not significantly different.

RT-qPCR analysis was performed to determine whether *Esi0017_0056* has any influence on the expression of several key stress responsive genes of *A. thaliana*. The expression of 12 genes including *DREB2A* (Dehydration-Responsive Element Binding Protein 2A)*, RD29A* (Responsive to Desiccation 29A)*, RD29B* (Responsive to Desiccation 29B), *RD26* (Responsive to Desiccation 26), *RD22* (Responsive to Desiccation 22), *RD20* (Responsive to Desiccation 20)*, RAB18* (Responsive to Abscisic Acid)*, LEA* (Late Embryogenesis Abundant)*, LEA14* (Late Embryogenesis Abundant 14), *NHX1* (Sodium/Hydrogen Exchanger), *HSP70* (Heat-Shock Protein 70) and *HSFA1D* (Heat Stress Transcription Factor A-1D)), which were demonstrated to play key roles in salinity and temperature stress tolerance, was analyzed in *Es*17-Ox2. The expression of 3 key genes (*DREB2A*, *RD29A* and *RD29B*) was also confirmed in *Es*17-Ox1 (Supplementary Figure S8). The relative expression of almost all these stress induced marker genes was rather similar in *Es*17-Ox2 and wild type plants in standard conditions, but significantly up-regulated in salinity stress, at both time points (24 and 120 h), in the plants of the transgenic line (Figure 6). In normal conditions, the only gene found to be strongly up-regulated in *Es*17-Ox2 *vs* wild type comparison was that of *HSP70*, coding a ubiquitous *A. thaliana* heat shock protein (Figure 6k). Under salinity stress, the expression of the transcription factor *DREB2A* (Dehydration-Responsive Element-Binding protein 2A) was found to be >2 times up-regulated, at both time points, in *Es*17-Ox2 *vs* wild type comparisons (Figure 6a). The expression of RD (Responsive to Desiccation) genes, *RD29B* and *RD26,* was found to be up-regulated >2 fold at both time points (Figure 6c,d, respectively), *RD29A* and *RD20* was up-regulated >2 fold only at the first time point (Figure 6b,f, respectively) while that of *RD22* was not different in *Es*17-Ox2 and wild type plants. *RAB18* (coding for a protein from the dehydrin family), *LEA* (Late Embryogenesis Abundant) and *LEA14* were also found to be significantly up-regulated in *Es*17-Ox2, at both time points, and this difference in expression was generally >2 fold higher (Figure 6g–i, respectively). *NHX1*, an Na^+^/H^+^ antiporter, and *HSP70* were determined to be significantly up-regulated in *Es*17-Ox2 only at 24 h (Figure 6j,k, respectively) while the expression of *HSFA1D*, a member of the Heat Stress Transcription Factor (Hsf) gene family, showed no notable differences at the two time points analyzed (Figure 6l). 

## 3. Discussion

Plant response to abiotic stress tolerance involves a complex network of genes and stress signaling pathways. Production of stress signaling molecules is followed by the activation of various molecular mechanisms to protect the plant against stress. In the last decades, tremendous progress has been made to identify and characterize novel gene functions in various photosynthetic organisms, including the plant model *A. thaliana* [15]. From these studies, a large number of genes involved in responses to abiotic stresses have been discovered and used to generate transgenic plants with improved tolerance to these stresses [15]. Several homologs of these genes have been studied and characterized in different crop plants. The aim of this study was to characterize *Esi0017_0056,* an unknown function gene from the brown algal model *Ectocarpus* sp. and to determine if this protein, which in *Ectocarpus* sp. can be induced by different stresses [13], can trigger similar responses in green land plants such as *A. thaliana*. Constitutive expression of heterologous proteins may have negative effects on growth of plants under standard conditions [16]. To test if there was any negative effect of *Esi0017_0056* constitutive expression on growth, transgenic lines under stress inducible promoter were also generated. The results showed that in seedlings grown under standard growth conditions, the 35S constitutive expression lines were associated with reduced root length while decreased biomass was determined in *Es*17-Ox1. In contrast, all the lines that utilized the *RD29A* stress inducible promoter performed similarly to the wild type. This trend was not anymore observed when plants were grown to maturity; no clear-cut differences could be observed between the two promoter lines. In normal conditions, qPCR analyses of mature plants indicated that more *Esi0017_0056* transcripts were produced in 35S lines as compared to RD29A lines; clearly, the difference in transcript abundance was not associated with protein quantity because the phenotype of transgenic plants did not reflect this considerable difference. 

Under salinity and temperature stress, both types of transgenic lines performed better than the wild type. Expression of *Esi0017_0056* in *A. thaliana* significantly improved plant tolerance to salinity and high temperature stress in all lines, irrespective of the promoter used. 

It is worth mentioning that under salinity conditions, similarly to the normal conditions, no positive correlation could be observed between the *Esi0017_0056* transcript abundance and phenotypic data or between the former condition and electrolyte leakage. We speculate that the elevated level of expression of *Esi0017_0056*, comparable to that of *actin* or better in constitutive expression lines, was already very high for the translational system, which reached a saturation or a plateau level. This process can be observed in many biochemical processes and explains the limited similarities, in certain experimental conditions, observed between transcriptomics and proteomics [17,18,19].

Since *Esi0017_0056* has no close relative in green plants, it is extremely difficult to indicate the mechanism through which the product of this gene determines the observed changes in the overall plant growth in normal conditions and how it contributes to the improved tolerance to abiotic stresses. In the current study, we analyzed the expression of several well characterized abiotic stress responsive transcriptional factors and genes regulated by them. In plants, ABA plays a crucial role to improve abiotic stress tolerance [20]. Several abiotic stress responsive genes require ABA for their activation and some not; this indicates the existence of both ABA-dependent and ABA-independent stress signal transduction pathways [21,22]. In plants, transcriptional regulation of abiotic stress responsive genes depends on the two major class of cis- acting elements found in promoter region of these genes. These elements are known as ABRE (ABA responsive elements) and DRE (Dehydration Responsive Elements). ABREs are known to participate in ABA dependent and DREs are known to be involved in ABA independent stress signal transduction pathways [23,24,25,26]. *A. thaliana RD26* gene encodes a NAC transcription factor that has been shown to localize in the nucleus and is induced by drought, salinity and ABA [27]. The promoter region of *RD26* has been shown to contain four ABRE, one MYC, two MYB and one DRE recognition sites [20,28]. *RD20* gene functions in ABA dependent stress signaling pathway [29] and has been shown as a direct target of *RD26* [27]. The expression of both these genes, i.e., *RD26* g and *RD20*, was determined to be significantly up-regulated in *Es*17-Ox2 plants as compared to the wild type plants (Figure 6d,f, respectively). *A. thaliana DREB2A* gene encodes a transcription factor which regulates the expression of genes induced by salinity and drought stress [30]. *DREB2A* contains an ERF/AP2 (ethylene responsive element binding factor/APETALA2) DNA binding domain. This domain regulates the expression of downstream genes by interacting with cis-element DRE in their promoter region [31,32]. *A. thaliana* plants overexpressing constitutively active form of *DREB2A* exhibited up-regulation of *RD29A*, *RD29B* and *LEA14,* suggesting that these genes are the direct target of *DREB2A*. Promoter region of these genes has been shown to carry DRE core motifs [33]. The expression of *DREB2A* (Figure 6a) and its abovementioned downstream genes (Figure 6b,c,i, respectively) was found to be significantly up-regulated in *Es*17-Ox2 plants in contrast to the wild type plants suggesting that *Esi0017_0056* may possibly modulate, through an unknown mechanism, the expression of *DREB2A.* In *A. thaliana*, *AREB1* gene encodes an ABRE binding protein which is a key transcription factor. *AREB1* is up-regulated by salinity stress and modulates the expression of ABRE dependent stress responsive genes involved in ABA signaling [34,35]. Overexpression of *AREB1* has been shown to up-regulate the expression of key ABA inducible stress responsive genes belonging to LEA class proteins *At3g17520* (encoding a group 3 LEA class protein), *RD29B/LTI65, RAB18* [36,37,38,39], and ABA regulated *RD20*, which encodes a calcium binding protein [29]. LEA proteins are involved in plant responses to abiotic stress tolerance by stabilizing the cellular membranes, redox homeostasis, nucleic acids and protein structures [40,41,42] and have been suggested to be direct targets of *DREB2A* [43]. In this study, the two LEA genes studied (Figure 6h,i) as well as *RD29B, RAB18* and *RD20* (Figure 6c,g,f, respectively) were found to be up-regulated in *Es*17-Ox2 plants. Moreover, reduced leakage of electrolytes in all *Esi0017_0056* transgenic lines suggest higher cellular membrane integrity in the transgenic plants compared to that in the wild type plants. 

Heat shock proteins (HSPs) act as chaperones playing essential roles in the proper folding and refolding of proteins as well in stabilizing their structure in presence of stress. In *A. thaliana, HSP70* (*At3g12580*) encodes a molecular *HSP70* chaperon which is involved in abiotic stress tolerance by preventing protein aggregation and helping in the refolding of proteins under stressful conditions [43]. The product of this gene is also involved in transport of unstable proteins to lysosomes or proteasomes for their degradation [44]. Expression of *HSP70* was found to be up-regulated in *Es*17-Ox2 under both normal and salinity stress conditions, at both the time points. HSP70 was reported to be present almost everywhere in the cell, including Golgi apparatus, cell wall, chloroplast, cytoplasm, cytosol, mitochondrion, plasma membrane and vacuolar membrane [45] (TAIR; https://www.arabidopsis.org; accessed on 26 January 2021). As the expression of this HSP70 (*At3g12580)* was found to be increased in the *Es*17Ox-2 line, it is quite possible that this heat shock protein is involved in a specific interaction with Esi0017_0056 which can include assistance for proper folding. Overexpression of *HSP70* has been shown to up-regulate the expression of *DREB2A* and of other target genes of *DREB2A* including *LEA 14, AIL* (group 3 LEA), *RD29A* and of *RD29B* [33,43], and to improve plant tolerance to high temperature, salinity and drought stress [44]. In the current study, the expression of all of these genes, i.e., *DREB2A, RD29A, RD29B, AIL, LEA14* and *HSP70,* was found to be significantly up-regulated in *Es*17-Ox2 plants as compared to the wild type plants, at both time points (Figure 6a–c,h,i,k). At least two main possible scenarios involving HSP70 and Esi0017_0056 can be envisaged: the first one assumes that Esi0017_0056 performs some catalytic activity in *A. thaliana* cells while the second one that *Ectocarpus* protein is inactive in the heterologous expression system. In the first situation the elevated levels of HSP70 are associated with its role as a chaperone, helping Esi0017_0056 to fold properly and, therefore, ensuring that Esi0017_0056 can perform its role, which presumably is an enzymatic activity. As mentioned before, Esi0017_0056 has a DUF1768 domain which based on its similarity with *E. coli* YbiA might exhibit N-glycosidase activity. In *E. coli,* YbiA is involved in riboflavin biosynthesis; however, the family that contains YbiA was included in the NADAR (NAD and ADP-ribose) superfamily which comprises proteins predicted to be involved in NAD-utilizing pathways, possibly using ADP-ribose derivatives as substrates. Nevertheless, based on structure modelling, the N-glycosidase activity is more likely to be present. The N-glycosidase activity, that is, the removal of N-linked oligosaccharides, can occur on a wide range of substrates including glycopeptides, glycoproteins and rRNA; therefore, the effects at cellular level cannot be predicted unless the substrate is identified. Nevertheless, removal of N-linked oligosaccharides is an important step on protein inactivation and subsequent degradation [46] which might be associated with increase expression of various heat shock proteins. This scenario explains well the expression pattern of *HSP70 (At3g12580)* and of *DREB2A, RD29A, RD29B, AIL, LEA14*, genes whose expression is up-regulated by HSP70, but what activity Esi0017_0056 performs in the cell remains to be elucidated. The second scenario posits that HSP70 participates in the folding of Esi0017_0056 which is not performing any activity in *A. thaliana* cells in the absence of a suitable substrate in this heterologous system. In this situation, the overall increased abundance of HSP70 is responsible for the observed effects in *A. thaliana* at phenotypic and molecular levels, i.e., increased tolerance to salt and temperature stress and changes in expression of a number of genes including of those aforementioned. Definitely, these propositions involving HSP70 are just two of the many possible scenarios as some direct and indirect interaction with other cytosolic proteins such as RD29A and B, RAB18 and LEA cannot be ruled out. Moreover, most proteins having the DUF1768 domain are of bacterial origin; therefore, it cannot be ruled out that this molecular pattern is perceived by *A. thaliana* as a non-self, foreign molecule, triggering additional responses, including defense responses, that await characterization. Clearly, future work including an “omics” (transcriptomics or proteomics) approach is needed to understand in depth the effects triggered by the expression of Esi0017_0056 in *A. thaliana.* Additionally, studies of the potential interaction between HSP70 (At3g12580) with Esi0017_0056 and resolving the crystal structure of Esi0017_0056 could contribute to the better understanding of the roles of this protein in *A. thaliana* and *Ectocarpus* sp. 

## 4. Materials and Methods

### 4.1. Ectocarpus *sp*. Growth Conditions and Gene Isolation

*Ectocarpus* sp. (Dilwyn) Lyngbye unialgal strain 32 (accession CCAP 1310/4, isolated in San Juan de Marcona, Peru) was cultured into a 10 L plastic tank filled with filtered and autoclaved natural seawater supplemented with Provasoli nutrient medium at a concentration of 10 mL/L. The tank was maintained at 14 °C with 14 h light/10 h dark cycle, and light intensity of 40 μmol.m^−2^s^−1^. The culture was air bubbled with filtered (0.22 μm filter) compressed air. The algal culture was exposed to salinity stress (1450 mM NaCl) for 6 h. After 6 h, the algal cultures were harvested using filtration, dried and immediately flash frozen into liquid nitrogen. Total RNA was extracted using the method described by [47] with slight modifications [48], treated with Turbo DNAse (Ambion Austin, TX, USA), and converted into cDNA using a SuperScript IV Reverse Transcriptase (Life Technologies, Saint-Aubin, Essonne, France). 

### 4.2. Bioinformatics Analysis

Prediction of transmembrane helices or domains in protein was carried out using HMMTOP v. 2.0 (http://www.enzim.hu/hmmtop/; accessed on 26 January 2021) and TMHMM v. 2.0 (https://services.healthtech.dtu.dk/; accessed on 26 January 2021). Secondary structure homology modelling was performed using PSIPRED [49] (http://bioinf.cs.ucl.ac.uk/psipred; accessed on 26 January 2021) and JPRED (http://www.compbio.dundee.ac.uk/jpred/; accessed on 26 January 2021). To gain more information about the folding and the tertiary structure of Esi0017_0056, protein structure, homology-modelling was carried out using SWISS-MODEL [50]. The best model (26.71% sequence identity) was built by ProMod v. 3 3.0.0 using the hypothetical protein ybiA from *E. coli* as a template (SMTL ID: 2b3w.1; structure solved by NMR). As the model generated by SWISS-MODEL covered only the DUF1768-domain, another tertiary structure homology-modelling was carried out using DMPfold [51]. The PDB files generated by ProMod3 v. 3.0.0 and DMPfold were visualized using iCn3D [52].

To assess the evolutionary relationship of Esi0017_0056, the amino acid sequence was compared with that of similar protein sequences retrieved from GenBank by running a blastp search. Alignments of selected protein sequences were performed using MUSCLE [53] implemented in MEGA X [54]. Sequences were trimmed to a total of 163 amino acid positions in the final dataset. The evolutionary history was inferred by using the Maximum Likelihood method and JTT matrix-based model [55]. A discrete Gamma distribution was used to model evolutionary rate differences among sites (5 categories (+*G*, parameter = 0.8733)). The analysis involved 71 sequences from different species and that of Esi0017_0056. Evolutionary analyses were conducted in MEGA X [54].

Prediction of eukaryotic protein subcellular localization was performed using TargetP 2.0, Signal P, and DeepLoc - 1.0 (https://services.healthtech.dtu.dk; accessed on 26 January 2021), PSORT and iPSORT (http://ipsort.hgc.jp; accessed on 26 January 2021), and WoLF PSORT (https://wolfpsort.hgc.jp; accessed on 26 January 2021).

### 4.3. Cloning and Expression of Recombinant Protein in E. coli 

To investigate the expression of Esi0017_0056 protein in *E. coli,* the entry clone was generated using High Fidelity Platinum *Taq* polymerase (Invitrogen, Mississauga, ON, Canada) with attB primers. The attB PCR product was cloned into pDONR221 using the BP Clonase^TM^ II Gateway^®^ (Gateway^®^ Technology with Clonase II, Invitrogen, Mississauga ON, Canada). The entry clone was then introduced into the pDEST17 (N-6xHis) vector using the LR Clonase^TM^ II Gateway^®^. The expression clone (Supplementary Figure S9a) was transformed into BL21 (DE3) cells. Cells were grown in LB medium containing carbenicillin (50 µg/mL) at 37 °C and 200 rpm on an orbital shaker for 2–3 h to an OD_600_ of 0.4. The cells were then induced using L-arabinose to a final concentration of 0.1 %, and 2% ethanol was added to the medium during induction. The cells were grown overnight at 16 °C with aeration. The cells were centrifuged at 5000× *g* at 4 °C and suspended in 1/10 volume of cold lysis buffer as described by [56]. Cells were sonicated on ice using a Qsonica probe sonicator (ThermoFisher Scientific, Mississauga, ON, Canada). Uninduced, induced cells, soluble fraction and pellet were mixed with sample buffer and were used for SDS-PAGE. The gel was stained using Coomassie Brilliant Blue R-250. 

### 4.4. Recombinant *Esi0017_0056* Protein Analysis by LC-MS/MS

The excised gel slices were processed for LC-MS/MS as described by [57], with minor modifications. The samples were transferred to a 300 µL HPLC vial and were subjected to analysis by LC-MS/MS on a VelosPRO orbitrap mass spectrometer (ThermoFisher Scientific, Mississauga, ON, Canada) equipped with an UltiMate 3000 Nano-LC system (ThermoFisher Scientific, Mississauga, ON, Canada). Chromatographic separation of the digests was performed on PicoFRIT C18 self-packed 75 µm × 60 cm capillary column (New Objective, Woburn, MA, USA) at a flow rate of 300 nl/min. MS and MS/MS data were acquired using a data-dependent acquisition method in which a full scan was obtained at a resolution of 30,000, followed by ten consecutive MS/MS spectra in both higher-energy collisional dissociation (HCD) and collision-induced dissociation (CID) mode (normalized collision energy 36%). Internal calibration was performed using the ion signal of polysiloxane at *m/z* 445.120025 as a lock mass. Raw MS data were analyzed using Proteome Discoverer 2.2 (ThermoFisher Scientific, Mississauga, ON, Canada). Peak lists were searched against all the available protein databases as well as the cRAP database of common contaminants (Global Proteome Machine Organization). Cysteine carbamidomethylation was set as a fixed modification, while methionine (Met) oxidation, N-terminal Met loss and phosphorylation on serine, threonine and tyrosine were included as variable modifications. A mass accuracy tolerance of 5 ppm was used for precursor ions, while 0.02 Da for HCD fragmentation or 0.6 Da for CID fragmentation was used for product ions. Percolator was used to determine confident peptide identifications using a 0.1% false discovery rate (FDR).

### 4.5. A. thaliana Seedlings Growth Conditions

*A. thaliana* seedlings were produced as described by [58]. In brief, Seeds of WT and transgenic *A. thaliana* lines were surface sterilized using 2% (*v/v*) NaOCl and stratified at 4 °C for two days. Seeds were placed on the plates containing half strength Murashige and Skoog (MS) medium (Phytotech, Lenexa, KS, USA), supplemented with 1% (*w/v*) sucrose and solidified with 0.4% (*w/v*) Phytagel (Sigma, Mississauga, ON, Canada). Plates were maintained at 22 °C with 16 h light/8 h dark cycle, with light intensity of 100 µmol.m^−2^s^−1^. Plants grown in these conditions henceforth be referred to as seedlings. 

### 4.6. GUS Expression Analysis in A. thaliana 

To generate the transformation vector, PMDC140 (C-GUS) [59] was obtained from ABRC (Columbus, OH, USA). Stop codon was removed from the target gene, and entry clone was generated as mentioned earlier. The entry clone was introduced into the PMDC140 as described earlier. The transformation vector (*Esi0017_0056*–GUS) (Supplementary Figure S9b) was transformed into *Agrobacterium* strain GV310 (pMB90) using the freeze and thaw method. The recombinant *Agrobacterium* strain carrying the gene of interest was transformed to flowering *A. thaliana* (L.) Heynh, ecotype Columbia (Col-0) plants, using the floral dip method, as described by [60]. Positive transformants were selected as described by [58] and allowed to self for 3 generations. Five days old seedlings of F3 generation produced as previously described were used for GUS expression analysis.

### 4.7. Transient Expression in Tobacco

To generate the transformation vector, pEarleyGate 103 (C-GFP-HIS) [61] was obtained from ABRC, and the entry clone used in the previous section was introduced as described earlier. *Agrobacterium* transformed with vector pEarlyGate103 (*Esi0017_0056*-GFP-HIS) (Supplementary Figure S9c) was grown at 28 °C in LB medium containing appropriate antibiotics until it reached an OD_600_ of 0.8. Cells were centrifuged, re-suspended in infiltration medium at a final OD_600_ of 0.1 and infiltrated in tobacco leaves as described by [62]. After two days, several leaves were excised, cut in small pieces and examined for GFP expression using a LSM meta 510 confocal microscope (Carl Zeiss, Mississauga, ON, Canada).

### 4.8. Expression of Esi0017_0056 in A. thaliana 

To generate the transformation vectors, pEarleyGate 100 (35S) [61] and promoter less Gateway vector pMCS:GW [63] were obtained from ABRC. RESPONSIVE TO DESICCATION 29A gene (*RD29A*, a stress inducible gene) promoter sequence, 1000 bp upstream of ATG initiation codon was isolated from wild type *A. thaliana* (Col-0) plants [64]. The sequence was isolated using a primer pair with the restriction sites *EcoR1* and *Stu1*. The vector and PCR product were digested and ligated using NEB enzymes (NEB, Mississauga, ON, Canada). The ligated vector was transformed into one shot CcdB survival 2T1R *E. coli* competent cells (Invitrogen, Mississauga, ON, Canada). The entry clone used for recombinant protein in *E. coli* was introduced into pEarleyGate 100 (35S) and pMCS:GW (RD29A) as described above. The transformation vectors 35S:*Esi0017_0056* and RD29A:*Esi0017_0056* (Supplementary Figure S9d,e, respectively) were transformed into *Agrobacterium* strain GV310 (pMB90), and transgenic plants were generated as described in the GUS expression analysis in *A. thaliana* section.

### 4.9. Selection of Transformants and Homozygotes 

Positive transformants were selected as described by [58]. In brief, the seeds collected from transformed plants were grown on plates containing half strength Murashige and Skoog (MS) medium (Sigma, Mississauga, ON, Canada), supplemented with 1% (*w/v*) sucrose and solidified with 0.4% Phytagel (*w/v*) and containing ammonium glufosinate 40 µg/mL. Plates were maintained at 22 °C in a 16-h light/8-h dark cycle. Seeds obtained from the positive lines were allowed to self for 5 generations. Segregation was tested in each generation by growing 100 seeds on half MS plates containing 40 µg/mL of ammonium glufosinate. Expression of the transgene was tested in these plants by RT-qPCR. Two independent lines with the 35S promoter, named as *Es*17-Ox1 and *Es*17-Ox2, and three independent lines with the stress inducible promoter (RD29A), named as *Es*17-A, *Es*17-B and *Es*17-C, were selected for further experiments. 

### 4.10. Salinity Stress Tolerance

Four days old uniform seedlings produced as described earlier were transferred on plates containing half strength MS medium and 100 mM NaCl. Root length was marked on the day of transfer, and on the 7th day, plates were scanned with a high-resolution scanner (Epson Expression 10000 XL, Epson, Markham, ON, Canada). Root length and the number of lateral roots per cm of primary root were measured using Image J software (Research Services Branch, NIH, Bethesda, MD, USA). Percent leaf chlorosis was visually estimated on 7th day after transfer. Nine-days-old plants were used to determine the fresh and dry weight. The experiment was repeated 3 times with 30 plants in each treatment in all experiments.

To assess the effect of salinity stress, plants were grown on Jiffy peat pellets (Jiffy, Shippagan NB, Canada). After 13 days of growth, uniform plants were selected and used in the experiment. Water-saturated peat pellets were left for 3 days without watering and then were irrigated with 200 mM NaCl (40 mL per plant) to reach a concentration of 100 mM. After five days of growth, plants were watered (20 mL per plant) at three days of interval for three weeks. Plants were photographed after three weeks, and biomass was recorded for individual plant. The experiment was repeated 3 times with 5 replicates in each treatment, in all experiments. The effect of salt stress on membrane intactness was estimated by recording the electrolyte leakage using SympHony SB70C (VWR, Mississauga, ON, Canada) conductivity meter as described by [65].

### 4.11. Temperature Stress Tolerance

The experiment was performed according to a method described by [58]. In brief, after 9 days of growth on plates containing half strength MS medium, seedlings were exposed to 40 °C for 24 h. The seedlings were then allowed to recover for one week under standard conditions, and biomass was recorded. The experiment was repeated 3 times with 30 plants in each treatment in all experiments. 

### 4.12. Real-Time Quantitative PCR of Key Stress Responsive Genes in Overexpression Line

The expression of 12 stress responsive genes was investigated in 3 independent biological replicates of wild type and overexpression line *Es*17-Ox2 (Supplementary Table S2). Plants were grown and treated as described earlier. Total RNA was extracted from samples using GeneJET plant RNA purification kit (Thermo Scientific, Mississauga, ON, Canada), treated with DNAse and converted into cDNA using the RevetAID cDNA Synthesis kit (Thermo Scientific, Mississauga, ON, Canada). The relative transcript levels were determined by RT-qPCR, using the gene specific primers and *actin2* as the endogenous control (Supplementary Table S2) on a StepOne Plus Real-Time PCR system (Applied Biosystems, Mississauga, ON, Canada), using iTaq SYBR Green mix (Bio-Rad, Mississauga, ON, Canada). The relative expression was calculated using the delta-delta Ct method, and transcript abundance was normalized to the individual with the lowest expression. The expression of some key genes was also confirmed in *Es*17-Ox1 line.

### 4.13. Statistical Analyses

Analysis of Variance (ANOVA) with a confidence level of 95%, followed by Tukey post hoc test with an error rate of 5%, was used to perform multiple mean comparisons. Statistical analyses were performed using Minitab 19.0 (Minitab LLC, State College, PA, USA).

## 5. Conclusions

The expression, for the first time, of the unknown function gene from brown alga *Ectocarpus* sp. in *A. thaliana* resulted in enhanced tolerance to high salinity and high temperature stress. Gene expression analysis revealed that the expression of several key stress markers genes involved in various functions such abscisic acid mediated abiotic stress tolerance, sodium sequestration, chaperon activities and membrane stability was up-regulated in transgenic plants. The protein fused with C-terminal tag was produced in both *A. thaliana* and *Nicotiana benthamiana.* These results suggest that brown algae represent a valuable source of important genes that can be used for generating transgenic land plants with improved tolerance to a wide range of abiotic stresses. 

## Data Availability

All datasets generated for this study are included in the article/Supplementary Material.

## Supplementary Materials

The Supplementary Material for this article can be found online at https://www.mdpi.com/1422-0067/22/4/1971/s1.
