# Supplementary material for: A Novel Protein from Ectocarpus sp. Improves Salinity and High Temperature Stress Tolerance in Arabidopsis thaliana
Source: Int J Mol Sci. 2021 Feb 17;22(4):1971. doi: 10.3390/ijms22041971 (PMC7922944; doi:10.3390/ijms22041971)
Supplement: Supplementary file 1 [file ijms-22-01971-s001.zip › Supplementary Figures and Table S2.pdf]

International Journal of
Molecular Sciences

MDPI

Supplementary Figures and Tables

Supplementary Figure S1. PsiPred predictions of the secondary structure [49]. (a) PsiPred sequence plot and (b) PsiPred cartoon with confidence of prediction. Models were generated at: http://bioinf.cs.ucl.ac.uk/psipred/; 30 November 2020.

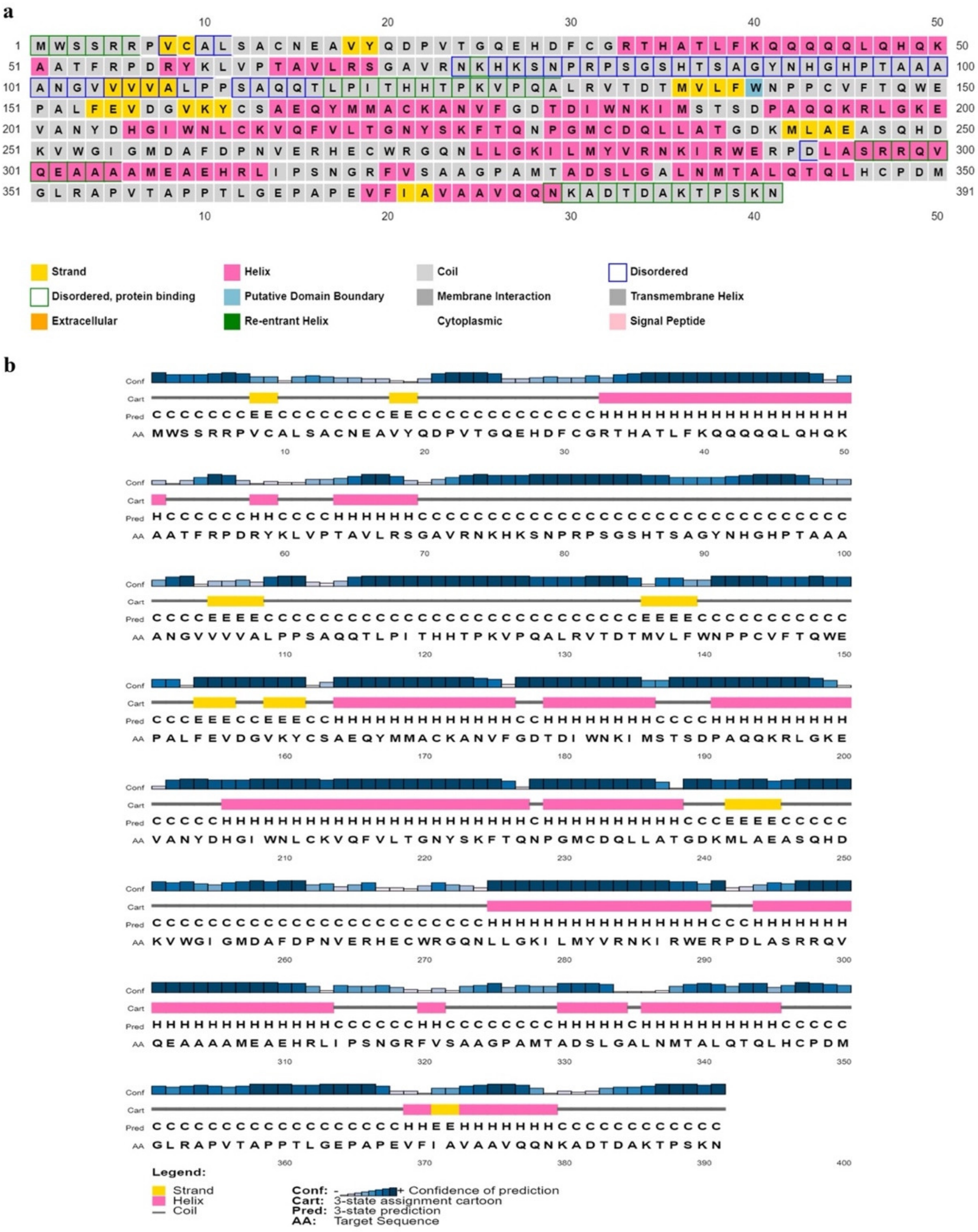

Supplementary Figure S2. Esi0017_0056 tertiary structure modelling. (a) Tertiary structure of the DUF1768-domain built with ProMod3 3.0.0 in SWISS-MODEL [50] using as template the hypothetical protein ybiA (SMTL ID: 2b3w.1.A); (b). The region covering residues 132 – 291 which was used to build the model, based on similarity with ybiA and (c). Tertiary structure of the full-length protein built by DMPfold [51]. The PDB files of the models generated by ProMod3 3.0.0 and DMPfold were visualized using iCn3D [52] as ribbon, spectrum colored models, at https://www.ncbi.nlm.nih.gov/structure (4 October 2020).

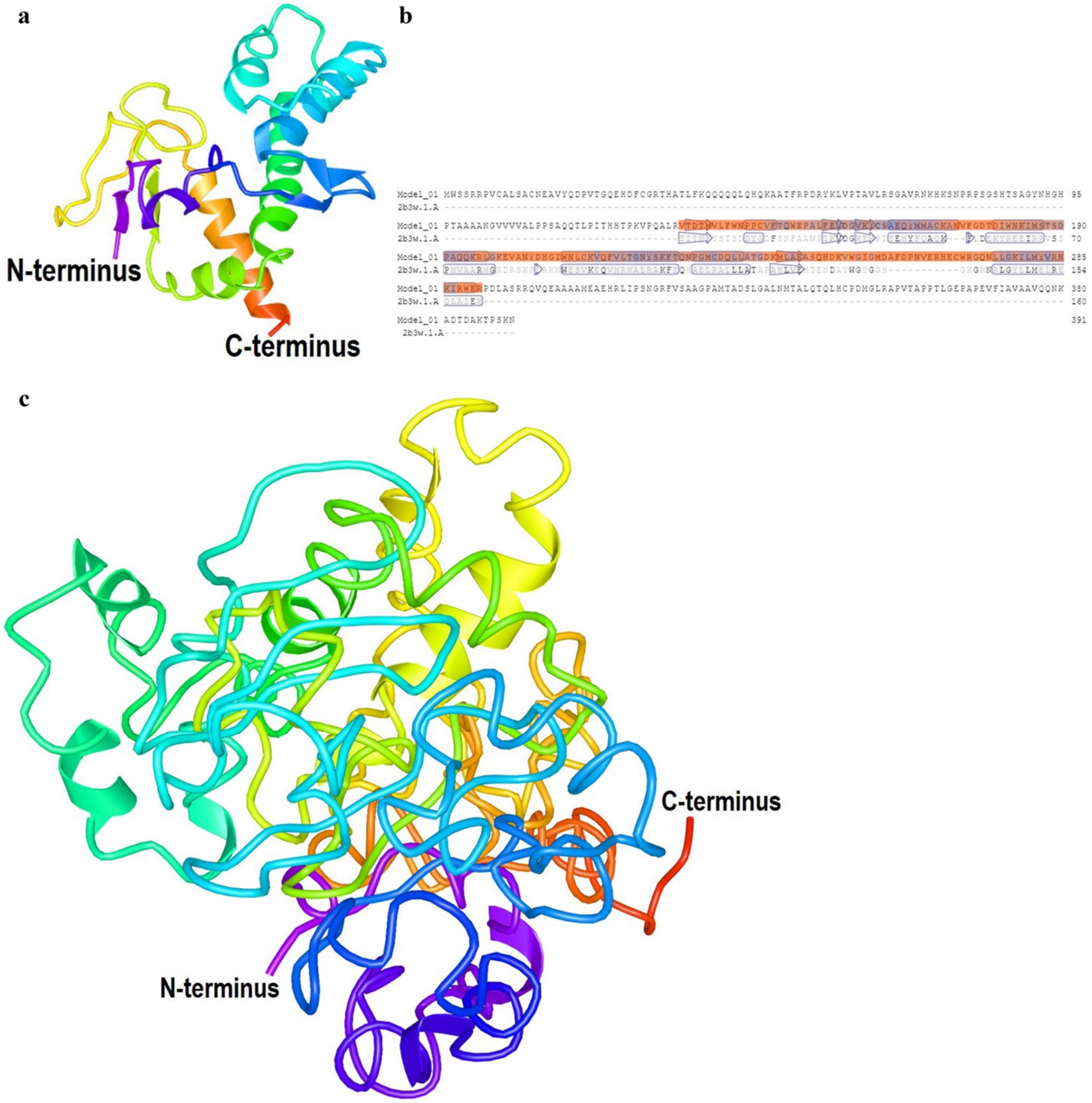

Supplementary Figure S3. Phylogenetic analysis of Esi0017_0056 protein using the Maximum Likelihood method and JTT matrix-based model. The percentage of trees in which the associated taxa clustered together is shown next to the branches.

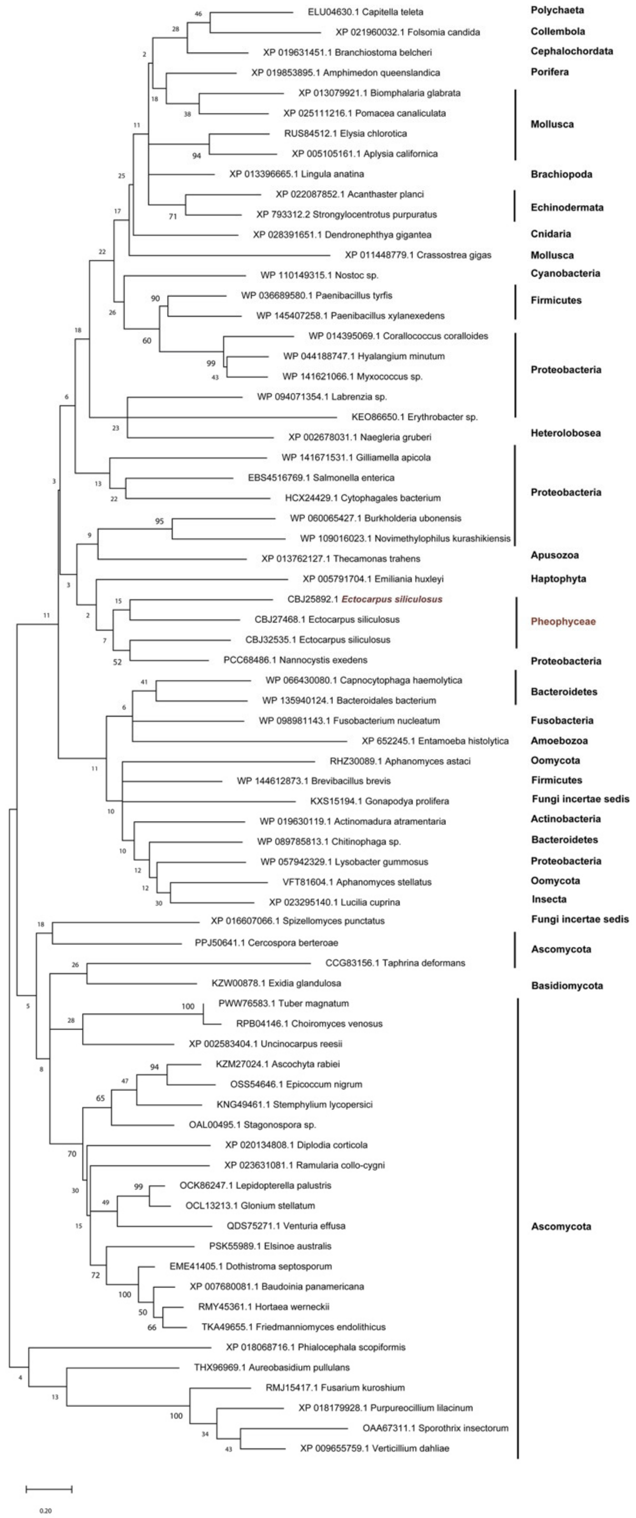

Supplementary Figure S4. Gene structure of *Esi0017_0056* indicating the presence of two introns.

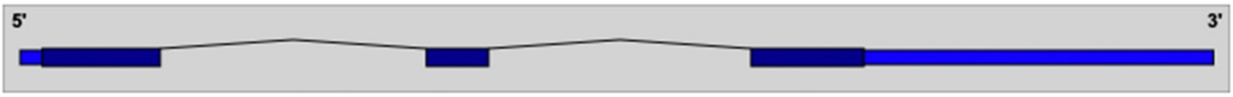

DOWNLOAD GENE IN EMBL FORMAT

**Structure**	558876..558964;558965..559438,560510..560758,561816..562268;562269..563675
**Sequence Type**	mRNA
**Strand**	+

Supplementary Figure S5. Expression of Esi0017_0056 protein with N-6xHis tag in *E. coli* BL21 DE3 cells. Lane 1, uninduced whole cells; lane 2, induced whole cells; lanes 3 to 5, supernatant of cell lysate; and lanes 6 to 8 pellet of cell lysate.

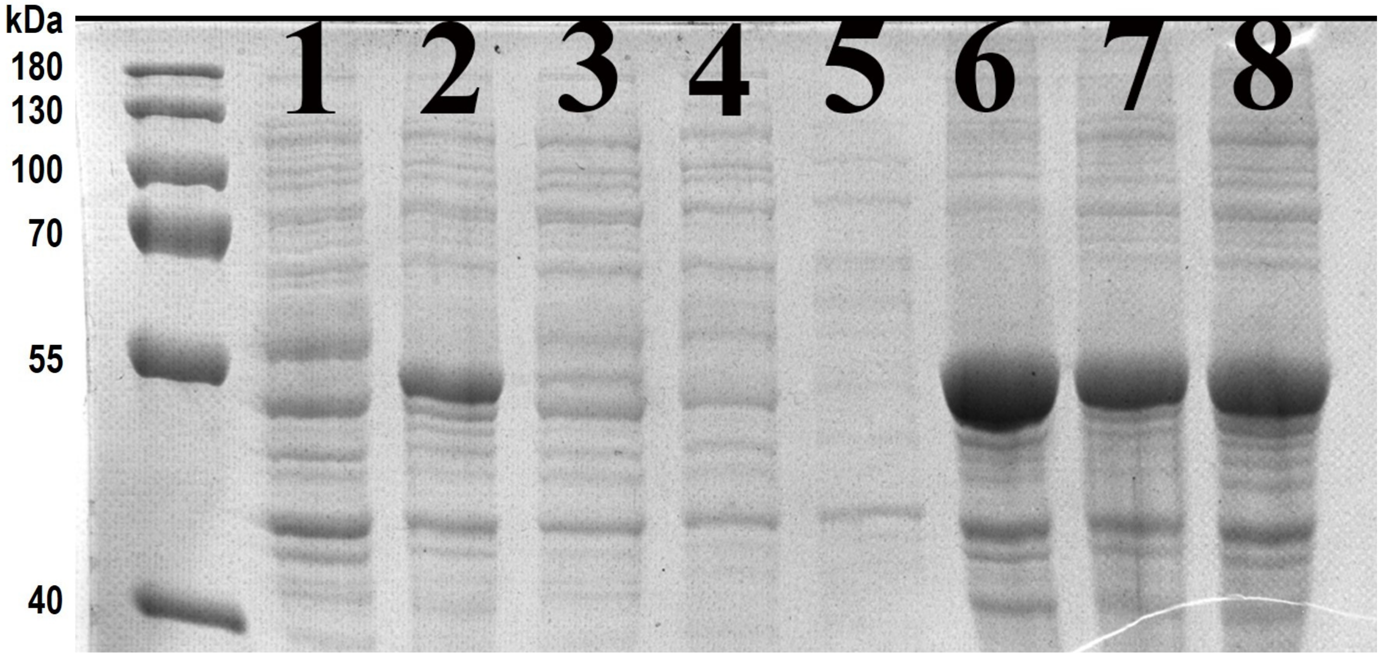

Supplementary Figure S6. Growth of wild type and 2 independent 35S promoter (*Es*17Ox-1-2,) and of 3 independent stress inducible promoter (*Es*17A-C) transgenic *A. thaliana* plants, expressing *Esi0017_0056*, in standard conditions. (a) WT, (b) *Es*17-A, (c) *Es*17-B, (d) *Es*17-C, (e) *Es*17-Ox1, (f) *Es*17-Ox2. The plants were photographed at 20 days after irrigation.

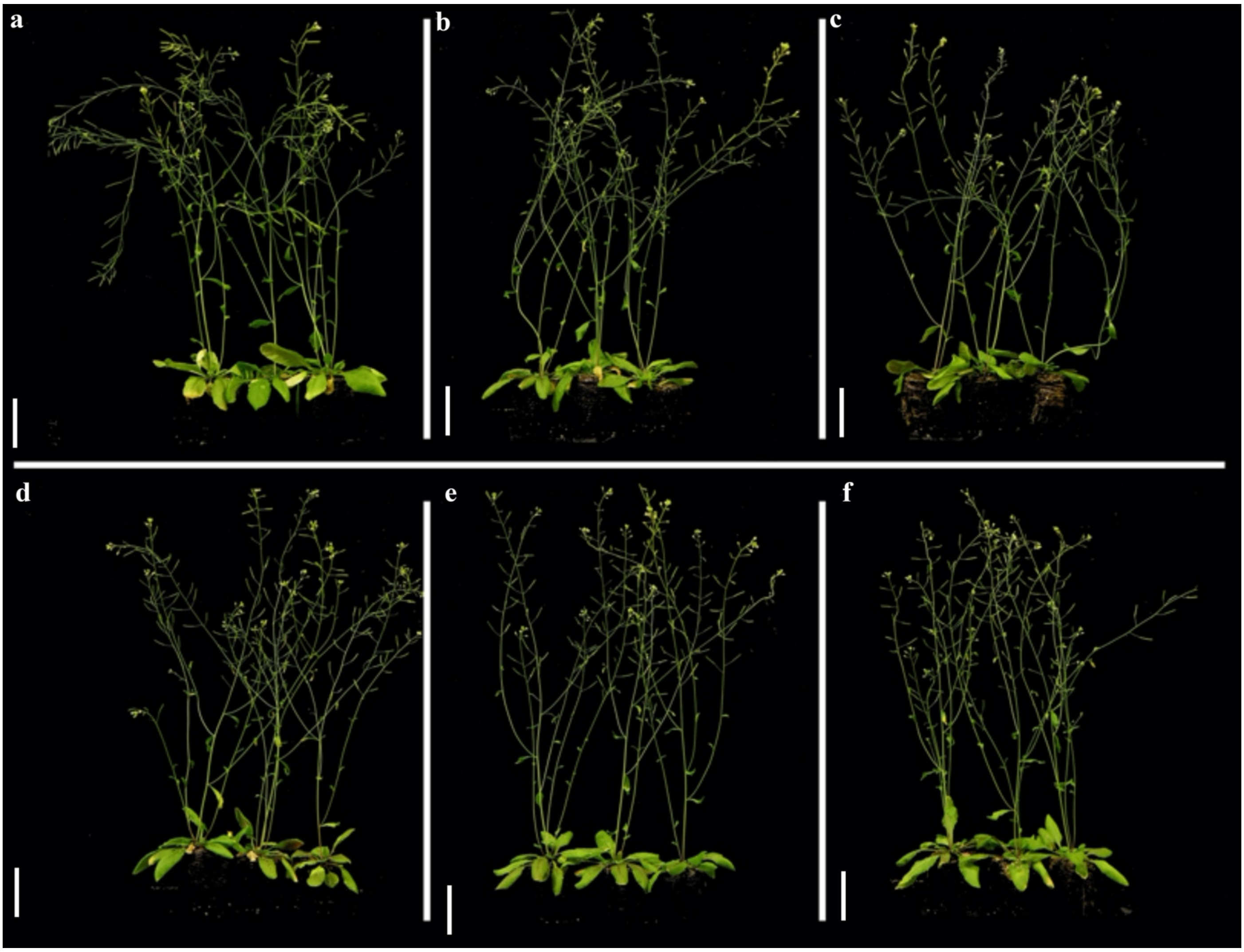

Supplementary Figure S7. Growth of wild type and 2 independent 35S promoter (*Es*17Ox-1-2,) and of 3 independent stress inducible promoter (*Es*17A-C) transgenic *A. thaliana* seedlings under high temperature stress and standard conditions. The seedlings in plates (a) to (f), were exposed to high temperature stress while seedlings in plates (g) to (l), were grown in standard conditions. (a, g) WT, (b, h) *Es*17-A, (c, i) *Es*17-B, (d, j) *Es*17-C, (e, k) *Es*17-Ox1, (f, l) *Es*17-Ox2, (m) Fresh weight and (n) dry weight. The 16 days old seedlings were photographed one week after being exposed to high temperature stress. Values represents mean and standard error (n=6). Means and SE followed by the same letter are not significantly different.

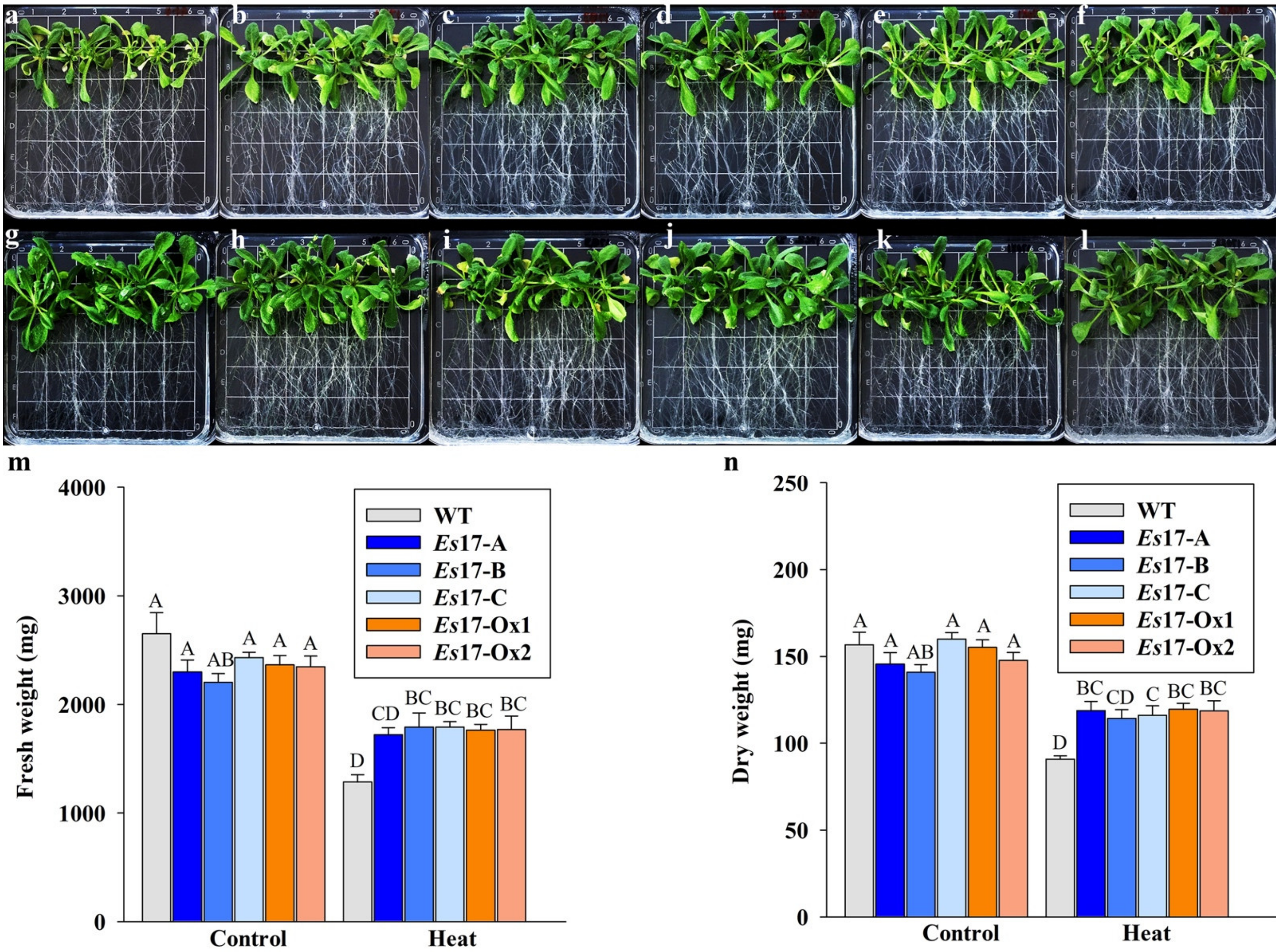

Supplementary Figure S8. Gene expression analysis of stress inducible marker genes in the wild type and transgenic *A. thaliana* plants (*Es*17Ox-1) grown in absence and presence of 100 mM NaCl. Two time points (24 and 120 h) were studied. *Actin* was used as the endogenous control and transcript levels were normalized to the individual with the lowest expression. Default font values represent up-regulation while italicized font values represent down-regulation. Data represents mean ± SE from 3 biological replicates. (a) *DREB2A,* (b) *RD29A,* (c) *RD29B.* Means and SE followed by the same letter are not significantly different.

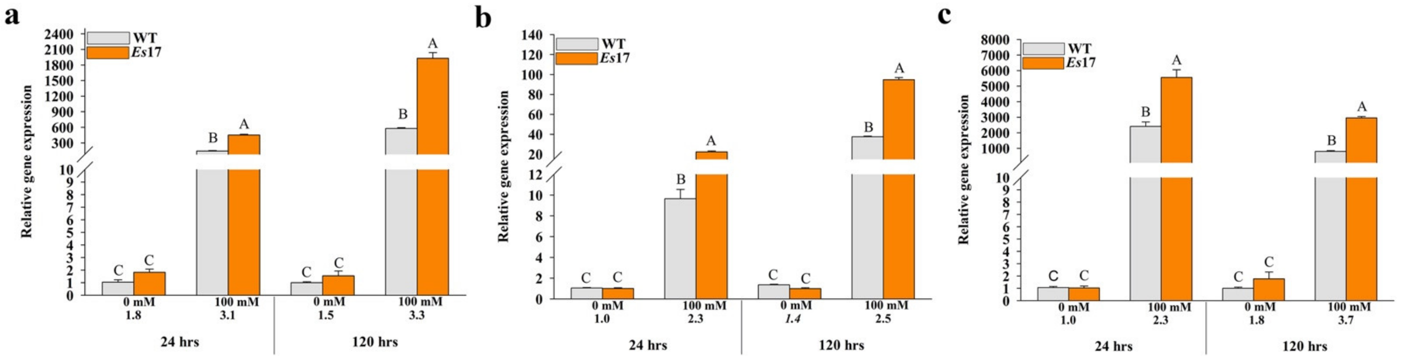

Supplementary Figure S9. Different constructs generated for expression of *Esi0017_0056* in plants and bacteria. (a) Expression under N-terminal 6xHis tag fusion. (b) C-terminal GUS fusion. (c) C-terminal GFP fusion. (d) Expression under 35S promoter in *A. thaliana*. (e) Expression under RD29A promoter in *A. thaliana*.

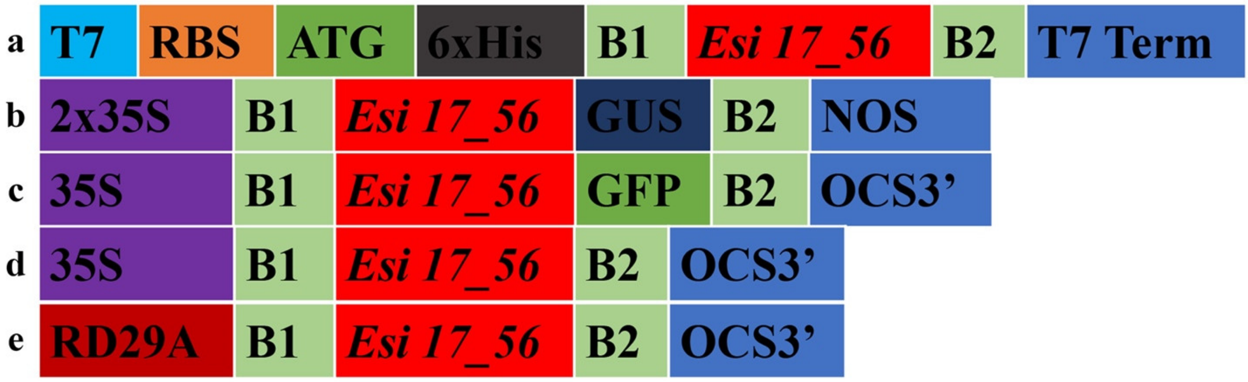

Supplementary Table S2. Primers used to amplify *A. thaliana* key marker genes involved in abiotic stress tolerance and *Ectocarpus* sp. unknown function gene *Esi0017_0056*.

Gene ID	Name	Sequence 5′ to 3′
AT3G18780	*Actin2* F	GCACCCTGTTCTTCTTACCG
	Actin2 R	AACCCTCGTAGATTGGCACA
AT5G05410	*DREB2A* F	TTGGCTGAGCGAGTTTGAAC
	DREB2A R	CGGTCCTGATTTAAGCCTGC
AT5G52310	*RD29A* F	TTTGGTGACGAGTCAGGAGC
	RD29A R	CACTACCAAAGCCCATCGGA
AT5G52300	*RD29B* F	CCAGAACTATCTCGTCCCAAAG
	RD29B R	GAAGCTAACTGCTCTGTGTAGG
AT4G27410	*RD26* F	TTGATTGGGCTAGCTTGGCA
	RD26 R	AGTTCTGCTGCCGATTCACA
AT5G25610	*RD22* F	TGGAGAAGGACTTGGTTCGC
	RD22 R	GAACCAGCTTCCACCGAGAA
AT2G33380	*RD20* F	TACACTTCCGAGTTGGGTGC
	RD20 R	AACCGTTAGCGCGTATTTGC
AT5G66400	*RAB18* F	GGGAGGAGGAAGAAGGGAATA
	RAB18 R	CGTAGCCACCAGCATCATATC
AT3G17520	*LEA* F	AGCTAAGGAAGCGGCTAAAC
	LEA R	CCTTAGCTGCACTCGTCATATC
AT1G01470	*LEA14* F	GGCCAAAGTCTCTGTCACCA
	LEA14 R	CTTTCAGAGAACCCGGGTCC
AT5G27150	*NHX1* F	GCCTCGGCCTGACAGTATAC
	NHX1 R	GTACAAAGCCACGACCTCCA
AT3G12580	*HSP70* F	CTTGGGTTTGGAAACTGCCG
	HSP70 R	GTTGTCCTTTGTTCGTGCCC
AT1G32330	*HSFA1D* F	CGAGCAAGCCAAAGCAATGT
	HSFA1D R	TCCATCTCTGTTCCCTCGGT
Esi0017_0056	*Esi17_56* F	CACGGCATTTGGAACCTGTG
	Esi17_56 R	GGATCAAATGCGTCCATGCC
