# Supplementary material for: A Novel Protein from Ectocarpus sp. Improves Salinity and High Temperature Stress Tolerance in Arabidopsis thaliana
Source: Int J Mol Sci. 2021 Feb 17;22(4):1971. doi: 10.3390/ijms22041971 (PMC7922944; doi:10.3390/ijms22041971)
Supplement: Supplementary file 1 [file ijms-22-01971-s001.zip › Supplementary Data S1.pdf]

CENTER FOR BIOLOGICAL SEQUENCE ANALYSIS ■ TECHNICAL UNIVERSITY OF DENMARK DTU

SignalP-5.0

Summary of 1 predicted sequences from Eukarya

Predictions list. Use the help page for more detailed description of the output page.

Predicted proteins

Sequence
Prediction: Other

Protein type	Signal Peptide (Sec/SPI)	Other
**Likelihood**	0.0167	0.9833

Download: PNG (/services/SignalP-5.0/tmp/5FDED0D00000654D26D5E92E/output_Sequence_plot.png) **/** EPS (/services/SignalP-5.0/tmp/5FDED0D00000654D26D5E92E/output_Sequence_plot.eps) **/** Tabular (/services/SignalP-5.0/tmp/5FDED0D00000654D26D5E92E/output_Sequence_pred.txt)

SignalP-5.0 prediction (Eukarya): Sequence

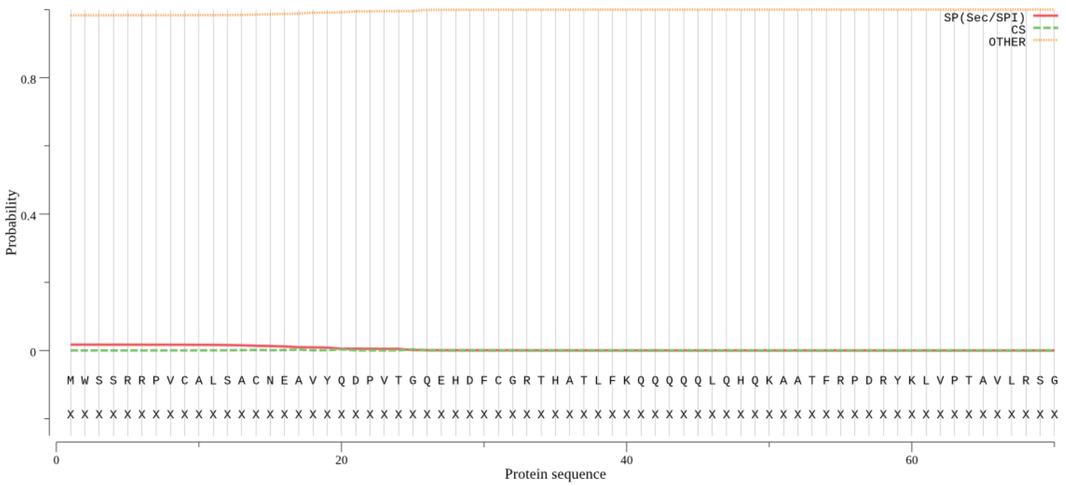

CENTER FOR BIOLOGICAL SEQUENCE ANALYSIS ■ TECHNICAL UNIVERSITY OF DENMARK DTU

TargetP-2.0

Summary of 1 predicted sequences from Plant

Predictions list. Use the help page for more detailed description of the output page.

Predicted proteins

Sequence
Prediction: Other

Protein type	Other	Signal peptide	Mitochondrial transfer peptide	Chloroplast transfer peptide	Thylakoid luminal transfer peptide
**Likelihood**	0.9807	0.0017	0.0095	0.0058	0.0024

Download: PNG (/services/TargetP-2.0/tmp/5FDED1B400001B3E44C81AD5/output_Sequence_plot.png) **/** EPS (/services/TargetP-2.0/tmp/5FDED1B400001B3E44C81AD5/output_Sequence_plot.eps) **/** Tabular (/services/TargetP-2.0/tmp/5FDED1B400001B3E44C81AD5/output_Sequence_pred.txt)

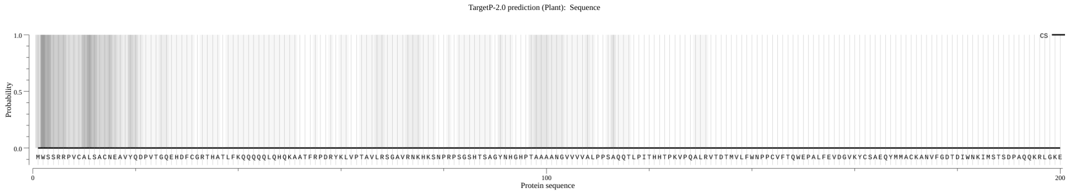

PSORT: Protein Subcellular Localization Prediction Tool

QUERY (391 aa)

```
MWSSRRPVCA LSACNEAVYQ DPVTGQEHDF CGRTHATLFK QQQQQLQHQK AATFRPDRYK

LVPTAVLRSG AVRNKHKSNP RPSGSHTSAG YNHGHPTAAA ANGVVVVALP PSAQQTLPIT

HHTPKVPQAL RVTDTMVLFW NPPCVFTQWE PALFEVDGVK YCSAEQYMMA CKANVFGDTD

IWNKIMSTSD PAQQKRLGKE VANYDHGIWN LCKVQFVLTG NYSKFTQNPG MCDQLLATGD

KMLAEASQHD KVWGIGMDAF DPNVERHECW RGQNLLGKIL MYVRNKIRWE RPDLASRRQV

QEAAAAMEAE HRLIPSNGRF VSAAGPAMTA DSLGALNMTA LQTQLHCPDM GLRAPVTAPP

TLGEPAPEVF IAVAAVQQNK ADTDAKTPSK N
```

Results of Subprograms

```
PSG:  a new signal peptide prediction method
      N-region:  length 6;  pos.chg 2;  neg.chg 0
      H-region:  length 9;  peak value   6.81
      PSG score:   2.41

GvH:  von Heijne's method for signal seq. recognition
      GvH score (threshold: -2.1):  -5.85
      possible cleavage site: between 20 and 21

>>> Seems to have no N-terminal signal peptide

ALOM: Klein et al's method for TM region allocation
      Init position for calculation: 1
      Tentative number of TMS(s) for the threshold  0.5:   0
      number of TMS(s) .. fixed
      PERIPHERAL  Likelihood =  2.17 (at 97)
      ALOM score:   2.17  (number of TMSs: 0)

MTOP: Prediction of membrane topology (Hartmann et al.)
      Center position for calculation: 6
      Charge difference: -6.5   C(-3.5) - N( 3.0)
      N >= C: N-terminal side will be inside

MITDISC: discrimination of mitochondrial targeting seq
      R content:       2       Hyd Moment(75): 10.51
      Hyd Moment(95):  8.53    G content:       0
      D/E content:     1       S/T content:     3
      Score: -1.37

Gavel: prediction of cleavage sites for mitochondrial preseq
      R-2 motif at 16  RRP|VC

NUCDISC: discrimination of nuclear localization signals
      pat4: none
      pat7: none
      bipartite: none
      content of basic residues:  10.2%
      NLS Score: -0.47

KDEL: ER retention motif in the C-terminus: none
```

ER Membrane Retention Signals:
      XXRR-like motif in the N-terminus: WSSR

      KKXX-like motif in the C-terminus: TPSK

SKL: peroxisomal targeting signal in the C-terminus: none

SKL2: 2nd peroxisomal targeting signal: none

VAC: possible vacuolar targeting motif: found
      TLPI at 116

RNA-binding motif: none

Actinin-type actin-binding motif:
      type 1: none
      type 2: none

NMYR: N-myristoylation pattern : none

Prenylation motif: none

memYQRL: transport motif from cell surface to Golgi: none

Tyrosines in the tail: none

Dileucine motif in the tail: none

checking 63 PROSITE DNA binding motifs: none

checking 71 PROSITE ribosomal protein motifs: none

checking 33 PROSITE prokaryotic DNA binding motifs: none

NNCN: Reinhardt's method for Cytplasmic/Nuclear discrimination
      Prediction: cytoplasmic
      Reliability: 55.5

COIL: Lupas's algorithm to detect coiled-coil regions
      total: 0 residues

Results of the *k*-NN Prediction

k = 9/23

      47.8 %: mitochondrial
      13.0 %: nuclear
      13.0 %: cytoplasmic
       8.7 %: endoplasmic reticulum
       8.7 %: Golgi
       4.3 %: extracellular, including cell wall
       4.3 %: vacuolar

>> prediction for 160843903028872 is mit (k=23)

iPSORT Prediction

Predicted as: *not having any of signal, mitochondrial targeting, or chloroplast transit peptides*

Sequence (Type: plant)

```
  1 MWSSR RPVCA LSACN EAVYQ DPVTG QEHDF CGRTH ATLFK QQQQQ LQHQK
 51 AATFR PDRYK LVPTA VLRSG AVRNK HKSNP RPSGS HTSAG YNHGH PTAAA
101 ANGVV VVALP PSAQQ TLPIT HHTPK VPQAL RVTDT MVLFW NPPCV FTQWE
151 PALFE VDGVK YCSAE QYMMA CKANV FGDTD IWNKI MSTSD PAQQK RLGKE
201 VANYD HGIWN LCKVQ FVLTG NYSKF TQNPG MCDQL LATGD KMLAE ASQHD
251 KVWGI GMDAF DPNVE RHECW RGQNL LGKIL MYVRN KIRWE RPDLA SRRQV
301 QEAAA AMEAE HRLIP SNGRF VSAAG PAMTA DSLGA LNMTA LQTQL HCPDM
351 GLRAP VTAPP TLGEP APEVF IAVAA VQQNK ADTDA KTPSK N
```

Values used for reasoning

Node	Answer	View	Substring	Value(s)	Plot
**1. Signal peptide?**	**No**	Average Hydropathy (KYTJ820101)	[6,25]	0.095 (>= 0.9225? No)	show
**2. Mitochondrial or chloroplast ?**	**No**	Average Negative Charge (FAUJ880112)	[1,30]	0.133333 (< 0.083? No)	show
		Indexing: AI1<br>Pattern: 22121222 (ins/del <= 2)	[1,30]	MWSSR-RPVCALSACNEAVYQDPVTGQEHDF<br>22221-1222222200222202220200002<br>22121222	--
**3. Mitochondrial?**	**No**	Average Isoelectric Point (ZIMJ680104)	[1,15]	6.396 (>= 6.21? Yes)	show
		Indexing: AI2<br>Pattern: 100100110 (ins/del <= 3)	[1,15]	-MW-SSRR-PVCALSACN<br>-00-0011-200000000<br>100100110	--

* This color means "not used".

Name	Alphabet Indexing		
	**0**	**1**	**2**
**AI1**	DEGHKN	IR	ACFLMPQSTVWY
**AI2**	ACDEFGHLMNQSTVWY	KR	IP

Return to iPSORT Home

160843885328766 WoLFPSORT prediction chlo: 6, cyto: 4, mito: 4

PSORT features and traditional PSORTII prediction

14 Nearest Neighbors

id	site	distance	identity	comments
ODPA_SOLTU	mito	259.787	11.75%	[Uniprot] SWISS-PROT45:Mitochondrial matrix.
GTH_SILCU	cyto	273.776	12.532%	[Uniprot] SWISS-PROT45:Cytoplasmic.
GLN2_CHLRE	chlo	282.603	12.4365%	[Uniprot] SWISS-PROT45:Chloroplast.
At2g30970.1	mito	285.134	12.761%	[Arath]
RBS1_CHLRE	chlo	287.271	12.0205%	[Uniprot] SWISS-PROT45:Chloroplast.
PODK_MAIZE	chlo	291.348	11.0876%	[Uniprot] SWISS-PROT45:Chloroplast.
ADH1_PETHY	cyto	291.367	8.39695%	[Uniprot] SWISS-PROT45:Cytoplasmic.
RBS5_ACEME	chlo	294.509	10.9974%	[Uniprot] SWISS-PROT45:Chloroplast.
FDH_SOLTU	mito	295.108	11.1959%	[Uniprot] SWISS-PROT45:Mitochondrial.
ADH3_SOLTU	cyto	302.578	11.2245%	[Uniprot] SWISS-PROT45:Cytoplasmic.
GLYM_FLAPR	mito	303.126	10.8317%	[Uniprot] SWISS-PROT45:Mitochondrial.
ADH2_LYCES	cyto	304.536	11.9898%	[Uniprot] SWISS-PROT45:Cytoplasmic.
RBS3_ACECL	chlo	304.855	10.9974%	[Uniprot] SWISS-PROT45:Chloroplast.
CB48_MAIZE	chlo	307.547	15.601%	[Uniprot] SWISS-PROT45:Chloroplast thylakoid membrane.

Normalized Feature Values

id	site	iPSORT		PSORT Features														Amino Acid Content								Misc.
		MxHy1_30	Mx-1_20	dna	erl	m1b	m3a	mNt	mip	mit	nuc	pox	psg	rib	rmp	tms	yqr	A	C	Q	H	I	L	S	V	length
160843885328766	chlo?	71	71	47	50	48	47	49	48	78	30	49	38	49	50	39	44	90	85	96	92	5	25	8	56	60
ODPA_SOLTU	mito	79	71	47	50	48	47	49	64	85	30	49	38	49	50	39	44	86	68	19	82	51	25	20	15	60
GTH_SILCU	cyto	49	71	47	50	48	47	49	55	71	30	49	38	49	50	39	44	75	13	84	97	12	95	4	56	26
GLN2_CHLRE	chlo	55	87	47	50	48	47	49	68	84	30	49	38	49	50	39	44	68	70	14	78	43	18	9	37	57
At2g30970.1	mito	66	87	47	50	48	47	49	66	78	30	49	38	49	50	39	44	58	54	72	89	35	48	56	26	65
RBS1_CHLRE	chlo	85	71	47	50	48	47	49	62	83	30	49	38	49	50	39	44	98	78	91	1	19	5	21	95	21
PODK_MAIZE	chlo	69	87	47	50	48	47	49	49	74	96	49	38	49	50	39	44	79	66	78	53	24	61	13	69	94
ADH1_PETHY	cyto	62	71	47	50	48	47	49	52	60	30	49	38	49	50	39	44	31	93	33	88	54	14	15	92	57
RBS5_ACEME	chlo	76	87	47	50	48	47	49	78	69	30	49	38	49	50	39	44	81	92	94	6	12	14	32	49	20
FDH_SOLTU	mito	52	71	47	50	48	47	49	63	88	30	49	38	49	50	39	44	82	49	42	88	66	63	4	37	57
ADH3_SOLTU	cyto	67	71	47	50	48	47	49	51	53	30	49	38	49	50	39	44	54	91	10	84	61	24	15	96	57
GLYM_FLAPR	mito	53	71	47	50	48	47	49	59	69	30	49	38	49	50	39	44	80	11	54	75	35	53	33	55	76
ADH2_LYCES	cyto	67	71	47	50	48	47	49	51	53	30	49	38	49	50	39	44	58	93	6	84	55	32	15	96	57
RBS3_ACECL	chlo	64	71	47	50	48	47	49	78	74	30	49	38	49	50	39	44	69	92	94	6	20	9	32	37	20
CB48_MAIZE	chlo	77	71	47	50	48	47	49	52	56	30	49	38	49	50	39	44	86	42	10	53	19	53	12	60	36

Raw Feature Values

id	site	iPSORT		PSORT Features														Amino Acid Content								Misc.
		MxHy1_30	Mx-1_20	dna	erl	m1b	m3a	mNt	mip	mit	nuc	pox	psg	rib	rmp	tms	yqr	A	C	Q	H	I	L	S	V	length
160843885328766	chlo?	13.50	2.00	0	0	0	0	0	16	-1.37	-0.47	0	-4	0	0	0	0	0.118	0.026	0.069	0.036	0.023	0.069	0.049	0.074	391
ODPA_SOLTU	mito	15.30	2.00	0	0	0	0	0	35	-0.07	-0.47	0	-4	0	0	0	0	0.110	0.018	0.020	0.028	0.054	0.069	0.059	0.054	391
GTH_SILCU	cyto	9.00	2.00	0	0	0	0	0	25	-2.61	-0.47	0	-4	0	0	0	0	0.097	0.005	0.046	0.051	0.032	0.120	0.042	0.074	216
GLN2_CHLRE	chlo	10.40	3.00	0	0	0	0	0	39	-0.30	-0.47	0	-4	0	0	0	0	0.092	0.018	0.018	0.026	0.050	0.063	0.050	0.066	380
At2g30970.1	mito	12.40	3.00	0	0	0	0	0	37	-1.50	-0.47	0	-4	0	0	0	0	0.084	0.014	0.040	0.033	0.047	0.084	0.079	0.060	430
RBS1_CHLRE	chlo	18.00	2.00	0	0	0	0	0	33	-0.39	-0.47	0	-4	0	0	0	0	0.157	0.022	0.054	0.000	0.038	0.049	0.059	0.103	185
PODK_MAIZE	chlo	13.10	3.00	0	0	0	0	0	17	-2.06	1.41	0	-4	0	0	0	0	0.101	0.017	0.042	0.019	0.041	0.091	0.054	0.080	947
ADH1_PETHY	cyto	11.80	2.00	0	0	0	0	0	21	-3.66	-0.47	0	-4	0	0	0	0	0.068	0.034	0.026	0.031	0.055	0.060	0.055	0.097	382
RBS5_ACEME	chlo	14.70	3.00	0	0	0	0	0	52	-2.89	-0.47	0	-4	0	0	0	0	0.104	0.033	0.060	0.005	0.033	0.060	0.066	0.071	183
FDH_SOLTU	mito	9.70	2.00	0	0	0	0	0	34	0.63	-0.47	0	-4	0	0	0	0	0.105	0.013	0.029	0.031	0.060	0.092	0.042	0.066	381
ADH3_SOLTU	cyto	12.70	2.00	0	0	0	0	0	20	-4.24	-0.47	0	-4	0	0	0	0	0.082	0.032	0.016	0.029	0.058	0.068	0.055	0.105	380
GLYM_FLAPR	mito	10.00	2.00	0	0	0	0	0	30	-2.87	-0.47	0	-4	0	0	0	0	0.103	0.004	0.033	0.025	0.046	0.087	0.066	0.074	517
ADH2_LYCES	cyto	12.70	2.00	0	0	0	0	0	20	-4.24	-0.47	0	-4	0	0	0	0	0.084	0.034	0.013	0.029	0.055	0.074	0.055	0.105	380

RBS3_ACECL	chlo	12.10	2.00	0	0	0	0	0	52	-2.06	-0.47	0	-4	0	0	0	0	0.093	0.033	0.060	0.005	0.038	0.055	0.066	0.066	183
CB48_MAIZE	chlo	14.90	2.00	0	0	0	0	0	21	-4.05	-0.47	0	-4	0	0	0	0	0.110	0.011	0.015	0.019	0.038	0.087	0.053	0.076	264

CENTER FOR BIOLOGICAL SEQUENCE ANALYSIS ▪ TECHNICAL UNIVERSITY OF DENMARK DTU

DeepLoc-1.0

Summary of 1 predicted sequences

Table of predicted subcelullar localizations. Use the help page for more detailed description of the output page.

Predicted proteins

Sequence
Prediction: Mitochondrion, Soluble

Localization	Mitochondrion	Cytoplasm	Extracellular	Nucleus	Plastid	Peroxisome	Lysosome/Vacuole	Cell membrane	Endoplasm reticulum
**Likelihood**	0.5139	0.2099	0.145	0.0637	0.036	0.0232	0.0027	0.0024	0.0024

Type	Soluble	Membrane
**Likelihood**	0.8906	0.1094

Hierarchical Tree. Donwload: PNG (/services/DeepLoc-1.0/tmp/5FDED13C00000345334C3C54/tree_1.png) **/** EPS (/services/DeepLoc-1.0/tmp/5FDED13C00000345334C3C54/tree_1.eps)

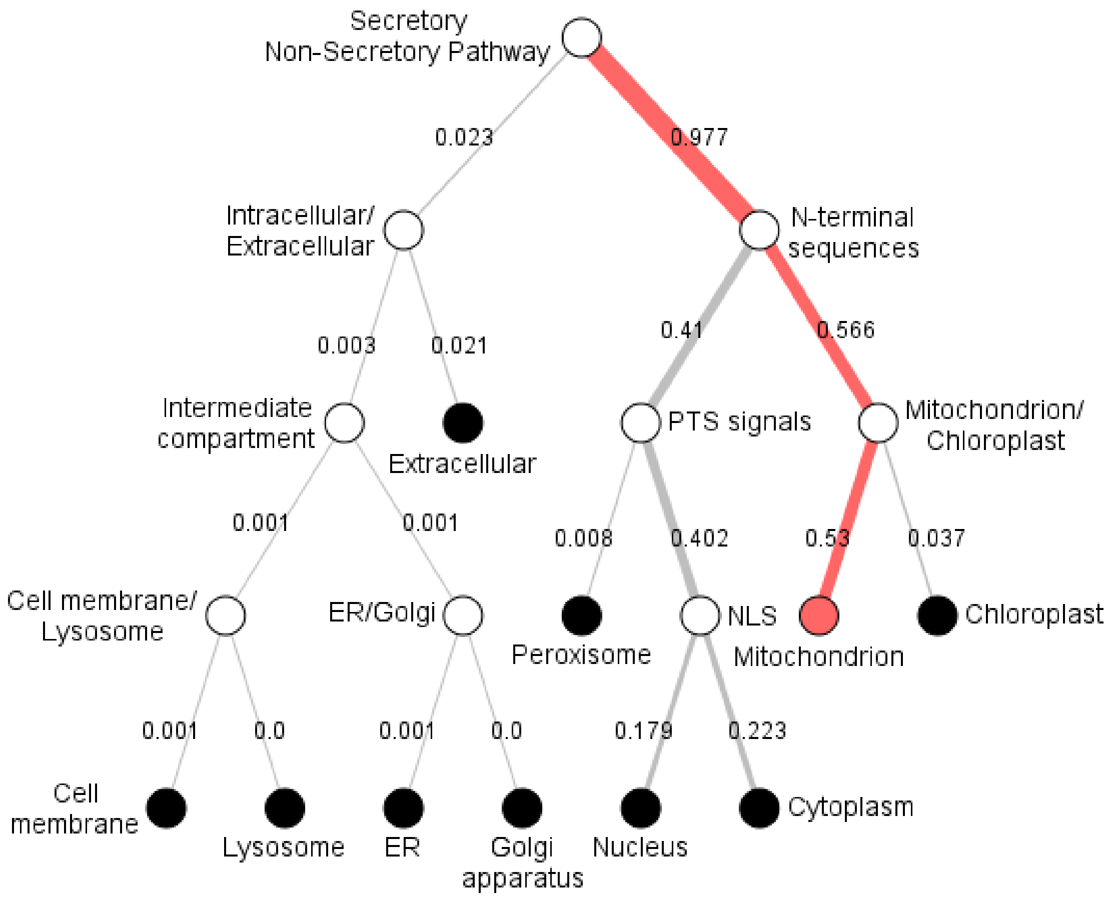

Position Importance. Donwload: PNG (/services/DeepLoc-1.0/tmp/5FDED13C00000345334C3C54/alpha_1.png) **/** EPS (/services/DeepLoc-1.0/tmp/5FDED13C00000345334C3C54/alpha_1.eps) **/** CSV (/services/DeepLoc-1.0/tmp/5FDED13C00000345334C3C54/alpha_1.csv)

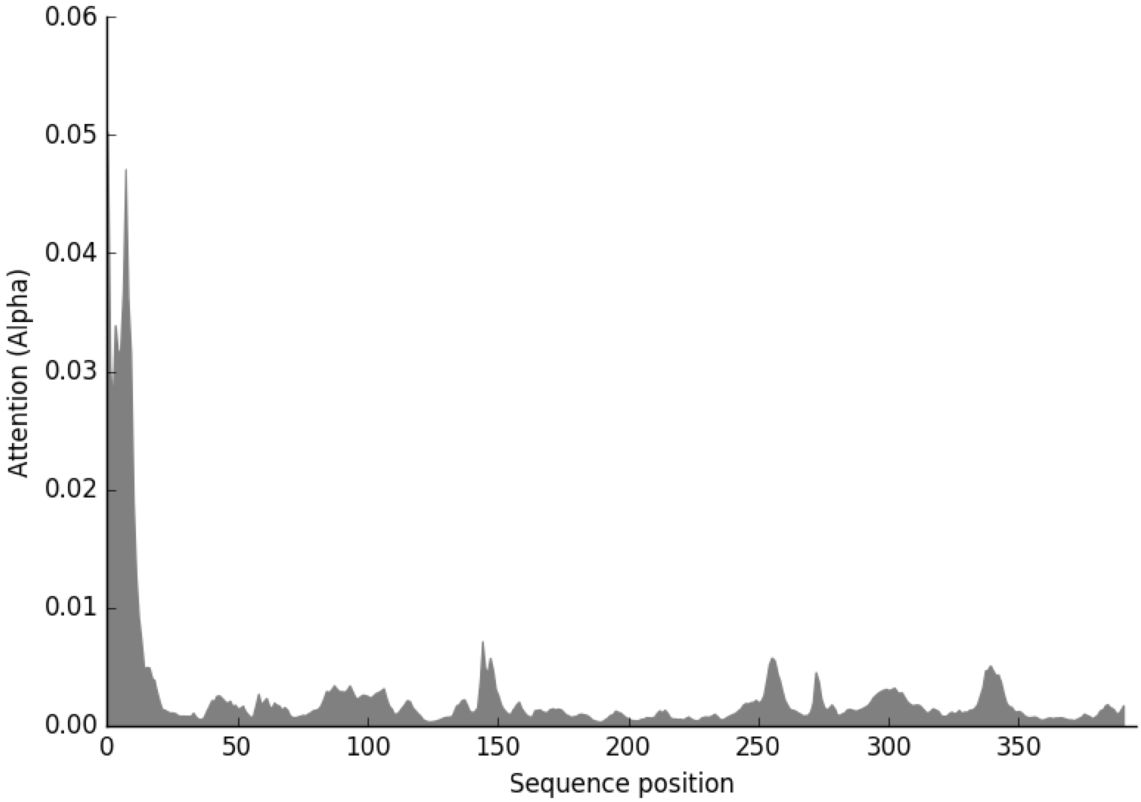
0.06
0.05
0.04
0.03
0.02
0.01
0.00
Attention (Alpha)
0
50
100
150
200
250
300
350
Sequence position
